# The Deubiquitinase OTUD1 Suppresses Secretory Neutrophil Polarization And Ameliorates Immunopathology of Periodontitis

**DOI:** 10.1002/advs.202303207

**Published:** 2023-08-28

**Authors:** Jia Song, Yuning Zhang, Yunyang Bai, Xiaowen Sun, Yanhui Lu, Yusi Guo, Ying He, Min Gao, Xiaopei Chi, Boon Chin Heng, Xin Zhang, Wenjing Li, Mingming Xu, Yan Wei, Fuping You, Xuehui Zhang, Dan Lu, Xuliang Deng

**Affiliations:** ^1^ Department of Geriatric Dentistry Peking University School and Hospital of Stomatology Beijing 100081 P. R. China; ^2^ Department of Dental Materials & Dental Medical Devices Testing Center Peking University School and Hospital of Stomatology Beijing 100081 P. R. China; ^3^ National Center for Stomatology National Clinical Research Center for Oral Diseases National Engineering Research Center of Oral Biomaterials and Digital Medical Devices NMPA Key Laboratory for Dental Materials Beijing Laboratory of Biomedical Materials & Beijing Key Laboratory of Digital Stomatology Peking University School and Hospital of Stomatology Beijing 100081 P. R. China; ^4^ Department of Orthodontics Peking University School and Hospital of Stomatology Beijing 100081 P. R. China; ^5^ Central Laboratory Peking University School and Hospital of Stomatology Beijing 100081 P. R. China; ^6^ Institute of Systems Biomedicine School of Basic Medical Sciences NHC Key Laboratory of Medical Immunology Beijing Key Laboratory of Tumor Systems Biology Peking University Health Science Center Beijing 100191 P. R. China; ^7^ Peking University‐Yunnan Baiyao International Medical Research Center Beijing 100191 P. R. China

**Keywords:** COPII, neutrophil, OTUD1, periodontitis, secretory machinery

## Abstract

Tissue‐infiltrating neutrophils (TINs) secrete various signaling molecules to establish paracrine communication within the inflammatory milieu. It is imperative to identify molecular mediators that control this secretory phenotype of TINs. The present study uncovers a secretory neutrophil subset that exhibits increased pro‐inflammatory cytokine production and enhanced migratory capacity which is highly related with periodontal pathogenesis. Further analysis identifies the OTU domain‐containing protein 1 (OTUD1) plays a regulatory role in this secretory neutrophil polarization. In human and mouse periodontitis, the waning of inflammation is correlated with OTUD1 upregulation, whereas severe periodontitis is induced when neutrophil‐intrinsic OTUD1 is depleted. Mechanistically, OTUD1 interacts with SEC23B, a component of the coat protein II complex (COPII). By removing the K63‐linked polyubiquitin chains on SEC23B Lysine 81, the deubiquitinase OTUD1 negatively regulates the COPII secretory machinery and limits protein ER‐to‐Golgi trafficking, thus restricting the surface expression of integrin‐regulated proteins, CD9 and CD47. Accordingly, blockade of protein transport by Brefeldin A (BFA) curbs recruitment of *Otud1‐*deficient TINs and attenuates inflammation‐induced alveolar bone destruction. The results thus identify OTUD1 signaling as a negative feedback loop that limits the polarization of neutrophils with secretory phenotype and highlight the potential application of BFA in the treatment of periodontal inflammation.

## Graphical abstract

Neutrophils lead the first wave of host innate immunity that rapidly migrate to inflamed site and exert effector functions. Here, it is identified that the deubiquitinase OTUD1 targets COPII secretory machinery and limits the polarization of neutrophils with secretory phenotype. Conversely, depletion of OTUD1 increases the susceptibility to periodontitis through augmenting the transport of proinflammatory cytokines and adhesive proteins on neutrophil.

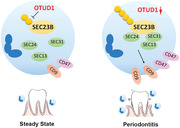

## 1 Introduction

As the most abundant leukocyte lineage in the subgingival crevice or periodontal pocket, tissue‐infiltrating neutrophils exacerbates various oral pathological conditions including recurrent gingivitis, periodontitis, candidiasis and oral ulcers.^[^ ^1^, ^2^ ^]^ Because of the diverse variety of inflammatory stimuli to which neutrophils respond, they must be able to adapt to various environments.^[^ ^3^ ^]^ Therefore, it has been observed that neutrophils adopt varying phenotypes under different inflammatory conditions. Moreover, the necessity for neutrophil recruitment under a wide variety of different conditions requires them to maintain phenotypic plasticity.^[^ ^4^ ^]^ This further supports the notion that neutrophils with varied functions are adapting to their environment and become differentially polarized, rather than starting off as a specific subtype. Despite accumulative evidences suggest that the various neutrophil populations should be regarded as subgroups of leukocytes, many of these subgroups lack distinctive markers and the categorization of neutrophil subpopulations is still a controversial topic.

While neutrophils have long been recognized to have prominent phagocytic functions involved in the clearance of pathogens and cell debris, increasing evidence of their crucial roles as essential communicators in shaping the immune response has emerged.^[^ ^5^ ^]^ The capacity of neutrophils to manipulate inflammatory and immune responses is dependent on their release of neutrophil‐derived molecules, including cytokines, alarmins, and neutrophil extracellular traps (NETs), as well as their capacity to interact with and direct other innate and adaptive immune cells.^[^ ^6^ ^]^ The coat protein complex II (COPII) is responsible for the secretion of neutrophil‐derived molecules from the endoplasmic reticulum (ER) to their final destination, either outside of the cell or on the cellular membrane system.^[^ ^7^ ^]^ A key component of COPII is SEC23, whose crucial role in maintaining cellular homeostasis is highlighted by the fact that mutations in the two SEC23 paralogs (SEC23A and SEC23B) cause the human genetic diseases cranio‐lenticulo‐sutural dysplasia and congenital dyserythropoietic anemia type II, respectively.^[^ ^8^, ^9^ ^]^ However, the role of the components of COPII vesicles in neutrophils and its regulatory mechanism are largely unknown.

In addition to proteasomal degradation of proteins, ubiquitination was identified to participate in receptor internalization and intracellular trafficking.^[^ ^10^ ^]^ For instance, monoubiquitination of the COPII‐component SEC31 by CUL3–KLHL12 controls the size of vesicle coat and facilitates secretion of much bigger cargos.^[^ ^11^ ^]^ As the deubiquitinase, the OTU domain‐containing protein 1 (OTUD1) has been reported to specifically hydrolyze ‘Lys‐63′‐linked polyubiquitin to monoubiquitin.^[^ ^12^ ^]^ Of note, mutant OTUD1 is detected in multiple autoimmune disorders including systemic lupus erythematosus (SLE), rheumatoid arthritis (RA) and ulcerative colitis (UC).^[^ ^13^, ^14^ ^]^ Accordingly, more effector cytokines were detected in serum from *Otud1*‐deficient mice upon viral infection.^[^ ^15^ ^]^ Recent studies revealed that OTUD1 is implicated in modulation of T cell or B cell‐mediated immune response.^[^ ^16^, ^17^ ^]^ However, the biological role of OTUD1 in neutrophils, especially its role in control of the secretion of neutrophils, is largely unknown.

In this study, we found that neutrophil secretory phenotype is associated with periodontal immunopathology and regulated by deubiquitinase OTUD1. Further analysis reveals that OTUD1 is mainly expressed by gingival neutrophils and selectively upregulated during periodontitis. Conversely, deletion of OTUD1 increases the susceptibility of mice to experimental periodontitis and exacerbates tissue damage. By targeting SEC23B, OTUD1 removes the K63‐linked polyubiquitin chain on SEC23B Lysine 81, thereby impairing the association of COPII components and subsequent protein trafficking. Furthermore, blockade of protein transport signaling by Brefeldin A (BFA) restricts *Otud1‐*deficient neutrophil recruitment and attenuates inflammation‐induced alveolar bone destruction. Our study thus explored the role of OTUD1 in moderating periodontitis by inhibiting neutrophil secretion, suggesting that the OTUD1‐SEC23B axis can be a potential target for the treatment of neutrophil‐related inflammatory diseases.

## 2 Results

### 2.1 Secretory Neutrophil Polarization is associated with Periodontal Immunopathology

**Figure 1:**
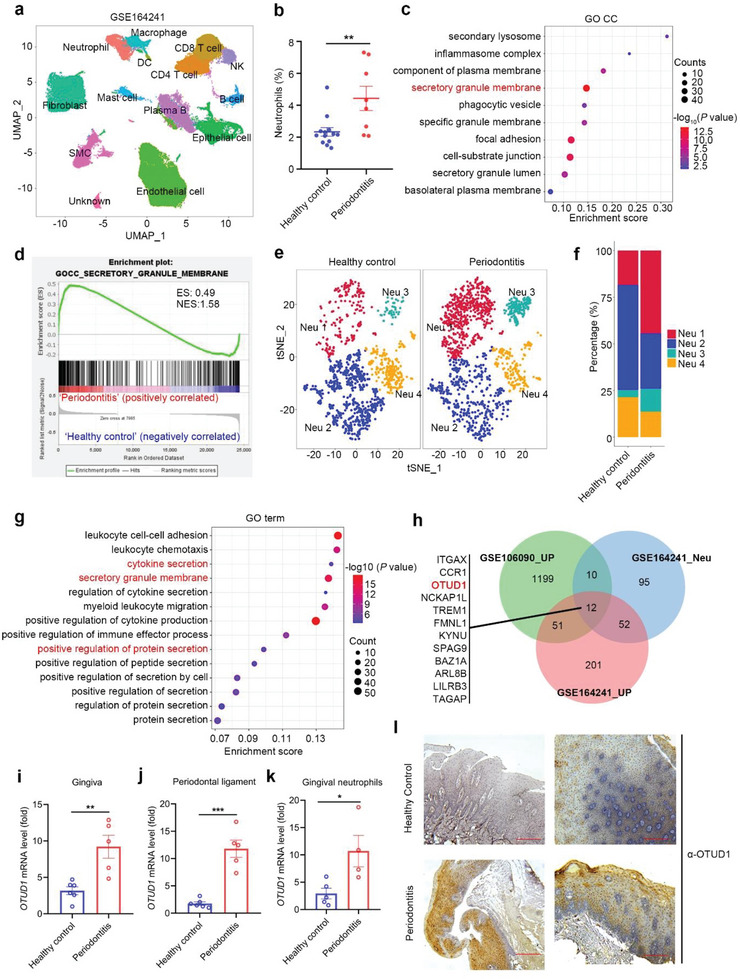
Neutrophil secretory phenotype is associated with periodontitis. a) Visualization of 13 cell clusters using UMAP, including neutrophils, CD4+ T cell, CD8+ T cell, B cells, plasma B cells, natural killer (NK) cells, macrophages, dendritic cells (DCs), mast cells, epithelial cells, endothelial cells, fibroblasts and smooth muscle cells (SMC) by analysis of data from GEO database (GSE164241). b) Proportion of gingival neutrophils in patients with periodontitis and healthy control by analyzing scRNA‐seq data (Healthy control, n = 13; Periodontitis, n = 8; mean ± s.e.m., **P = 0.0064, two‐tailed unpaired Student's t‐test). c) Genes upregulated in neutrophils of patients with periodontitis were analyzed with Gene Ontology (GO_CC) terms. d) GSEA of genes expressed in neutrophils of patients with periodontitis and healthy subjects. ES, enrichment score; NES, normalized enrichment score. e) t‐SNE plot showing single neutrophils of 4 distinct cell types (different colors), including Neu 1, Neu 2, Neu 3, and Neu 4 in patients with periodontitis and healthy control. f) Proportions of the 4 different neutrophil subpopulations in patients with periodontitis and healthy control are shown. g) Genes upregulated in Neu 1 of patients with periodontitis were analyzed with GO terms. h) Venn diagram analysis of GSE106090 and GSE164241 datasets. Green, protein coding genes upregulated in periodontitis patients from GSE106090 dataset (FC > 1.2, *P* < 0.05). Blue, neutrophil specific genes from GSE164241 dataset (FC > 1.2, PCT1 > 20%, PCT2 < 20%, *P* < 0.05). Red, genes upregulated in periodontitis patients from GSE164241 dataset (FC > 1.2, PCT1 > 20%, PCT2 < 20%, *P* < 0.05). i,j) Real‐time quantitative PCR (RT‐qPCR) analysis of *OTUD1* mRNA level in gingiva (i) and periodontal ligament (j) from patients with periodontitis and healthy control (Healthy control, n = 6; Periodontitis, n = 5; mean ± s.e.m., **P = 0.0035, ***P = 0.000078, two‐tailed unpaired Student's t‐test). k) Real‐time quantitative PCR (RT‐qPCR) analysis of *OTUD1* mRNA level in gingival neutrophils from patients with periodontitis and healthy control (Healthy control, n = 5; Periodontitis, n = 4; mean ± s.e.m., *P = 0.0255, two‐tailed unpaired Student's t‐test). l) Representative immunohistochemistry staining images of OTUD1 protein levels in gingivae from patients with periodontitis and healthy control. The scale bars represent 100 µm.

Periodontitis is a dysbiotic inflammatory disease characterized by dysregulation of periodontal immune response. For systematic assessment of immune status in periodontitis, we interrogated published single cell sequencing (scRNA‐seq) data from healthy adults and patients with periodontitis.^[^ ^18^ ^]^ We clustered all cells jointly in the Uniform Manifold Approximation and Projection (UMAP) space and annotated 13 major cell types by canonical marker genes (**Figure** **1**a; Figure S1A, Supporting Information). Conceivably, the proportion of neutrophil was significantly increased in patients with periodontitis (Figure 1b). By comparing differentially expressed genes in neutrophils between the two groups, several inflammation‐associated genes, such as *IL1B*, *CD44*, *CXCL8*, and *CCL4* were upregulated in the gingivae from patients with periodontitis versus healthy subjects (Figure S1B,C, Supporting Information). Further analysis revealed that genes upregulated in neutrophils from periodontitis patients were mainly related with the biological processes of “secretory granule membrane” (Figure 1c,d). We next defined four heterogeneous neutrophil subpopulations, designated as Neu 1, Neu 2, Neu 3, and Neu 4, respectively (Figure 1e). As shown in Figure 1f, the ratio of Neu1 was significantly increased in patients with periodontitis. Compared with other subsets of neutrophil, Neu1 preferentially expressed genes related with immune effector processes, such as *IL1A*, *CD44*, *CCL20*, and *NLRP3* (Figure S2A, Supporting Information). Accordingly, genes associated with the GO terms “cytokine secretion”, “protein secretion”, and “receptor complex” were overrepresented in the significantly upregulated genes in Neu 1 from patients with periodontitis, relative to those in healthy control (Figure 1g; Figure S2B, Supporting Information). We thus designated this subset of neutrophils as the secretory neutrophils.

In order to find the key immune regulator of neutrophil secretory phenotype, we jointly investigated neutrophil specific genes upregulated in periodontitis from microarray (GSE106090) and scRNA‐seq (GSE164241) data. 12 eligible candidates were yielded and the deubiquitinase OTUD1 is of interest (Figure 1h). We next experimentally assessed the status of OTUD1 during periodontitis. As shown in Figure 1i–l, both transcription level and protein level were remarkably increased in patients gingival tissues and gingival neutrophils with periodontal inflammation, supporting the notion that OTUD1 is upregulated during the pathogenesis of periodontitis. Together, these findings thus reinforce the critical role of secretory neutrophils in periodontitis and OTUD1 might be involved in modulation of periodontal immune response.

### 2.2 Inflammation‐Responsive OTUD1 plays a Suppressive Role in Periodontal Pathogenesis

**Figure 2:**
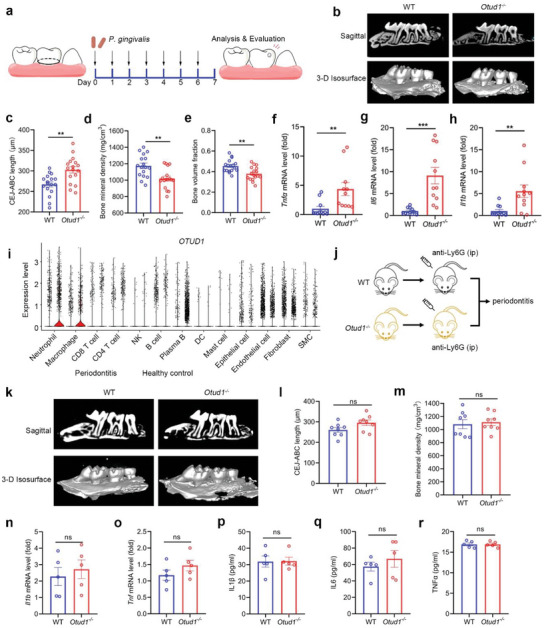
Loss of OTUD1 aggravates periodontal inflammation. a) Schematic illustration of the generation of experimental periodontitis by ligature placement and *Porphyromonas gingivalis* (*P. gingivalis*) infection. b) Representative micro‐CT sagittal and 3‐D Isosurface images of maxillary alveolar bone surrounding the second molar 7 days after periodontitis induction. c) Analysis of the cemento‐enamel junction‐alveolar bone crest (CEJ‐ABC) distance of the second molar obtained by micro‐CT (n = 17, mean ± s.e.m., **P = 0.0012, two‐tailed unpaired Student's t‐test). d) Bone mineral density around the second molar was analysed by micro‐CT imaging (n = 17, mean ± s.e.m., **P = 0.0039, two‐tailed unpaired Student's t‐test). e) Bone volume fraction around the second molar was assessed by micro‐CT imaging (n = 17, mean ± s.e.m., **P = 0.0015, two‐tailed unpaired Student's t‐test). f–h) RT–qPCR analysis of indicated pro‐inflammatory cytokine mRNA levels in gingival tissues from WT and *Otud1* ^─/─^ mice after periodontitis induction (n =  11, mean ± s.e.m., **P = 0.0100 (*Tnfα*), **P  =  0.0059 (*Il1β*), ***P = 0.0003, two‐tailed unpaired Student's t‐test). i) Violin plot showing the expression levels of *OTUD1* in different clusters by scRNA‐seq data from patients with periodontitis and healthy control. j) A schematic illustration of the neutrophil depletion assay. WT or *Otud1* ^─/─^ mice were intraperitoneally injected with anti‐Ly6G antibody to delete neutrophils. Experimental periodontitis induction was then performed. k) Representative micro‐CT sagittal and 3D Isosurface images of maxillary alveolar bone surrounding the second molar after periodontitis induction in the context of neutrophil depletion. l‐m) Micro‐CT analysis of CEJ‐ABC distance and bone mineral density (BMD) around the second molar from WT or *Otud1* ^─/─^ mice with neutrophil depletion (n = 8; mean ± s.e.m., ns, not significant (*P* > 0.05), two‐tailed unpaired Student's t‐test). n,o) RT–qPCR analysis of *Il1β* and *Tnf* mRNA levels in gingival tissues from WT and *Otud1* ^─/─^ mice with neutrophil depletion after periodontitis induction (n = 5, mean ± s.e.m., ns, not significant (*P* > 0.05), two‐tailed unpaired Student's t‐test). p–r) Protein expression levels of indicated pro‐inflammatory cytokines within mice serum in the context of experimental periodontitis (n = 5, mean ± s.e.m., ns, not significant (*P* > 0.05), two‐tailed unpaired Student's t‐test).

To further investigate the biological role of OTUD1 during the process of periodontitis, we utilized *Otud1* ^−/−^ or WT mice and induced experimental periodontitis by ligature placement and *Porphyromonas gingivalis* (*P. gingivalis*) infection (**Figure** **2**a). The hallmark of periodontitis is inflammation‐mediated alveolar bone loss.^[^ ^19^ ^]^ Compared with WT littermates, *Otud1* ^−/−^ mice exhibited more severe periodontal bone loss, along with increased cemento‐enamel junction‐alveolar bone crest (CEJ‐ABC) distance, decreased bone mineral density and bone volume fraction (Figure 2b–e). Additionally, we found that both mRNA and protein levels of pro‐inflammatory cytokines were increased in the gingiva and serum from *Otud1* ^−/−^ mice as compared with those from WT mice (Figure 2f–h; Figure S3A,B, Supporting Information). Collectively, our data demonstrate that loss of inflammation‐responsive OTUD1 increases the susceptibility of mice to periodontitis.

### 2.3 Neutrophil‐Intrinsic OTUD1 is Essential for its Inhibitory Effects on Periodontal Inflammation

While OTUD1 is known to be ubiquitously expressed in multiple tissues, the biological functions of OTUD1 are manifested in a context‐dependent manner, depending on the specific tissue, organ or pathological condition.^[^ ^14^ ^]^ To determine whether loss of OTUD1 in immune cells or non‐immune cells contributes to severe periodontitis, we generated bone marrow chimeric mice via adoptive transfer of bone marrow. Following periodontitis induction, the group of WT mice receiving *Otud1* ^−/−^ bone marrow (*Otud1* ^−/−^→WT) exhibited more severe symptoms of periodontitis than those in the WT auto‐transplantation (WT→WT) group, which were determined by the CEJ‐ABC length, bone mineral density as well as increased amounts of inflammatory cytokines (Figure S3C–I, Supporting Information). Reciprocally, no significant difference was found between WT and *Otud1* ^−/−^ mice receiving WT bone marrow (Figure S3J–P, Supporting Information). These data thus demonstrated that immune cell‐intrinsic OTUD1 is responsible for mediating resistance to ligature and *P. gingivalis* induced periodontitis.

In light of the critical role of neutrophil in tissue inflammation and OTUD1 expression profile (Figure 2i), we thus hypothesized that OTUD1 exerted its regulatory effects on neutrophil. To further confirm this, we used anti‐Ly6G antibody to eliminate neutrophils and induced mice periodontitis. Subsequent histological and immunological assessments showed that neutrophil‐depleted WT and *Otud1* ^−/−^ mice exhibited similar susceptibility to periodontitis (Figure 2j–r). Moreover, we employed clodronate liposomes to eliminate macrophages and induced mice periodontitis and subsequent histological and immunological assessments showed that macrophage‐depleted *Otud1* ^−/−^ mice still exhibited severe alveolar bone loss and periodontal inflammation (Figure S3Q–V, Supporting Information). Our findings thus indicate that loss of OTUD1 in neutrophils contributes to the severe inflammatory damage.

### 2.4 Loss of OTUD1 Promotes the Polarization of Neutrophil with Secretory Phenotype

**Figure 3:**
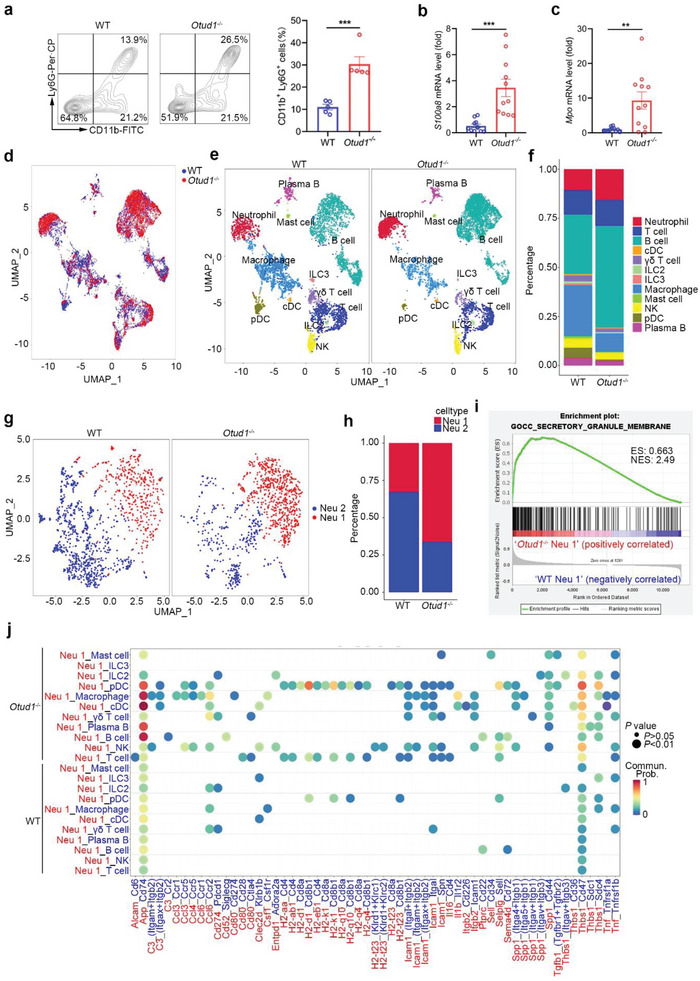
OTUD1 limits the polarization of neutrophils with secretory phenotype. a) Flow cytometric analysis of neutrophils (CD11b^+^Ly6G^+^ cells) in WT and *Otud1* ^─/─^ mice gingiva after periodontitis induction (n = 5, mean ± s.e.m., ***P = 0.0006, two‐tailed unpaired Student's t‐test). b‐c) RT–qPCR analysis of mRNA levels of neutrophil‐related genes within gingival tissues of WT and *Otud1* ^─/─^ mice after periodontitis induction (n = 11, mean ± s.e.m., **P = 0.0033, ***P = 0.0004, two‐tailed unpaired Student's t‐test). d–f) CD45^+^ immune cells were isolated from the gingiva of WT and *Otud1* ^─/─^ mice after periodontitis induction, followed by 10× single cell RNA‐sequencing (scRNA‐seq). UMAP plot shows the single CD45^+^ immune cells in WT and *Otud1* ^─/─^ mice. WT, blue; *Otud1* ^─/─^, red (d). UMAP plot showed the single immune cells of 12 distinct lineages distinguished by different colors, including neutrophils, T cells, B cells, cDCs, γδ T cells, ILC2s, ILC3s, macrophages, mast cells, NK cells, pDCs and plasma B cells (e). Proportions of the 12 different cell types in WT and *Otud1* ^─/─^ mice gingiva are shown (f). g,h) UMAP plot showing single neutrophils of 2 distinct cell types (different colors), including Neu 1 and Neu 2 (g). Proportions of the 2 neutrophil subsets in WT and *Otud1* ^─/─^ mice gingiva are shown (h). i) GSEA of genes expressed in *Otud1* ^─/─^ Neu 1 and WT Neu 1. ES, enrichment score; NES, normalized enrichment score. j) Bubble plots showing ligand‐receptor pairs between secretory neutrophils (Neu 1) and other cell groups. Potential ligand‐receptor pairs between cell groups were predicted by CellChat. Red, ligands; blue, receptors.

To study the mechanism by which deletion of OTUD1 exacerbates periodontitis, we performed a series of assays including flow cytometry, quantitative real‐time PCR, immunohistochemistry and routine blood test to assess the status of neutrophils during periodontitis. As shown in **Figure** **3**a–c; Figure S4A (Supporting Information), increased amounts of gingiva‐infiltrated neutrophils were detected in *Otud1* ^−/−^ mice during periodontitis. In contrast, the proportion of neutrophils in peripheral blood was decreased in *Otud1* ^−/−^ mice compared with WT mice, while there is no significant difference in the bone marrow (Figure S4B–E, Supporting Information).

To further analyze the characteristics of neutrophils in *Otud1* ^−/−^ mice during periodontitis, we employed scRNA‐seq on the 10× Genomics platform to investigate the immune microenvironment in gingiva within the context of experimental periodontitis. We defined 12 clusters based on the exclusive expression of canonical marker genes for transcriptomic data including 17 995 single cells (Figure 3d; Figure S4F, Supporting Information). As shown in Figure 3e, the major immune cell types in gingiva includes neutrophils, T cells, B cells, γδ T cells, macrophages, plasmacytoid dendritic cells (pDCs), conventional dendritic cells (cDCs), type 2 innate lymphoid cells (ILC2s), type 3 innate lymphoid cells (ILC3s), mast cells, natural killer (NK) cells and plasma B cells. Consistent with the flow cytometric data, the percentage of neutrophils was increased in *Otud1* ^−/−^ mice (Figure 3f). We also observed increased percentage of B cells and reduced ratio of macrophages in *Otud1* ^−/−^ mice, as compared with WT mice (Figure 3f). We next performed unsupervised clustering and identified two clusters for neutrophils with its unique signature genes according to the 10× scRNA‐seq data (Figure 3g). The first cluster of neutrophils (Neu 1) preferentially expressed genes encoding pro‐inflammatory cytokines, including *Il1α*, *Il1β*, and *Tnfα* (Figure S5A, Supporting Information). Ensuing Gene Ontology (GO) analysis revealed that genes related to protein secretion, granulocyte migration and chemotaxis were enriched in Neu 1 as compared with Neu 2 (Figure S5B, Supporting Information). Using RNA velocity analysis, embedded on a diffusion map to infer the future fate of cells, we identified a strong directional flow from Neu 2 toward Neu 1, which triggers pro‐inflammatory response (Figure S5C, Supporting Information). As expected, the percentage of secretory neutrophils (Neu 1) was significantly increased in *Otud1* ^−/−^ mice (Figure 3h). To determine whether the secretory neutrophil subsets induced by OTUD1 depletion is similar to Neu1 from patients with periodontitis, we analyzed the status of genes altered in neutrophil subsets and found that the Neu1 subset in mice exhibited similar characteristics to those of the secretory neutrophil subset in Neu 1 from patients with periodontitis (Figure S5D,E, Supporting Information). Of note, we analyzed the differentially expressed genes in the secretory neutrophils between WT and *Otud1* ^−/−^ mice. As shown in Figure 3i, the enrichment of genes related to the secretory granule membrane was more pronounced in *Otud1* ^−/−^ secretory neutrophils, as compared with the WT controls. Our data thus indicate that loss of OTUD1 promotes the development of neutrophil with secretory phenotype during periodontitis.

To assess the role of *Otud1* ^−/−^ secretory neutrophils in regulating inflammatory milieus, we analyzed cell‐cell communications between secretory neutrophils and other immune cells based on the relative abundance of ligand‐receptor pairs. The results showed that more pronounced interaction of *Otud1* ^−/−^ secretory neutrophils with other immune cells such as cDCs, B and T cells was detected, relative to their WT controls (Figure 3j). Taken together, our data thus demonstrate that loss of OTUD1 augments oral inflammatory response by inducing secretory neutrophil polarization and provide insights into cell functionality and intercellular interactions in disease pathogenesis.

### 2.5 OTUD1 Limits the Inflammatory and Migratory Capacities of Neutrophil

**Figure 4:**
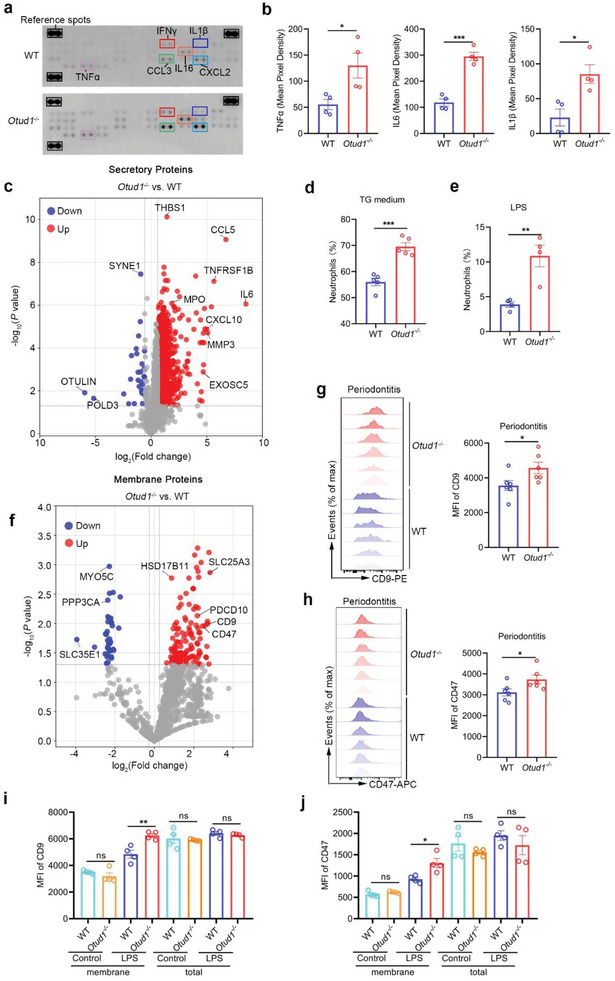
OTUD1 depletion enhances the presentation of surface molecules in neutrophils. a,b) Secretory cytokine array was performed to detect multiple analytes within cell culture supernatant of neutrophils from WT and *Otud1* ^─/─^ mice upon LPS (100 ng ml^−1^) stimulation. Quantitative analysis of average relative expression levels of indicated secretory cytokines was shown (n = 4, mean ± s.e.m., *P < 0.05, ***P = 0.0001, two‐tailed unpaired Student's t‐test). c) Volcano plots analysis of the secretory proteins in neutrophils between WT and *Otud1* ^−/−^ mice. Red, proteins upregulated in *Otud1* ^−/−^ neutrophils versus WT neutrophils. Blue, proteins downregulated in *Otud1* ^−/−^ neutrophils versus WT neutrophils. d‐e) Flow cytometric analysis of intraperitoneal neutrophils (CD11b^+^Ly6G^+^ cells) in WT and *Otud1* ^─/─^ mice after peritonitis induction by TG medium (n = 5, mean ± s.e.m., ***P = 0.0002, two‐tailed unpaired Student's t‐test) and LPS (n = 4, mean ± s.e.m., **P = 0.0048, two‐tailed unpaired Student's t‐test). f) Bone marrow derived neutrophils from WT and *Otud1* ^─/─^ mice were isolated and stimulated with LPS (100 ng ml^−1^). Neutrophil membranes were collected and quantitative proteomics analysis was performed. Volcano plots analysis of the membrane proteins in neutrophils between WT and *Otud1* ^−/−^ mice was performed. Red, proteins upregulated in *Otud1* ^−/−^ neutrophils versus WT neutrophils. Blue, proteins downregulated in *Otud1* ^−/−^ neutrophils versus WT neutrophils. g) Flow cytometric analysis of CD9 expression levels in gingiva infiltrated neutrophils in WT and *Otud1* ^─/─^ mice after periodontitis induction (n = 6, mean ± s.e.m., *P = 0.0442, two‐tailed unpaired Student's t‐test). h) Flow cytometric analysis of CD47 expression level in gingiva infiltrated neutrophils in WT and *Otud1* ^─/─^ mice after periodontitis induction (n = 6, mean ± s.e.m., *P = 0.0438, two‐tailed unpaired Student's t‐test). i) Flow cytometric analysis of membrane or total CD9 expression level in bone marrow derived neutrophils in WT and *Otud1* ^─/─^ mice upon LPS (100 ng ml^−1^) stimulation (n = 4, mean ± s.e.m., ns, not significant (*P* > 0.05), **P = 0.0059, two‐tailed unpaired Student's t‐test). j) Flow cytometric analysis of membrane or total CD47 expression level in bone marrow derived neutrophils in WT and *Otud1* ^─/─^ mice upon LPS (100 ng ml^−1^) stimulation (n = 4, mean ± s.e.m., ns, not significant (*P* > 0.05), *P = 0.0189, two‐tailed unpaired Student's t‐test).

Besides *P. gingivalis* infection, lipopolysaccharide (LPS) can also trigger neutrophil activation, adhesion and release of pro‐inflammatory cytokines. In order to evaluate the role of OTUD1 in the secretory functions of neutrophil, we isolated the neutrophils from WT and *Otud1* ^−/−^ bone marrow and stimulated them with LPS. Through analysis of expression levels of multiple inflammatory cytokines within the supernatant of WT and *Otud1* ^−/−^ neutrophils, we found that effector cytokines such as TNFα, IL6, IL1β, and CCL3, were significantly increased in *Otud1* ^−/−^ neutrophils as compared with those in WT neutrophils (**Figure** **4**a,b; Figure S6A, Supporting Information). Furthermore, we collected secretory proteins from cell culture supernatant of activated WT and *Otud1* ^−/−^ neutrophils. From the quantitative mass spectrometry analysis, we found the amount of secretory proteins was significantly increased in *Otud1* ^−/−^ neutrophils (Figure 4c). Similar result was observed in neutrophils treated with LPS derived from *P. gingivalis* (LPS‐PG) (Figure S6B, Supporting Information).

To further confirm this result in vivo, WT and *Otud1* ^−/−^ mice were intraperitoneally injected thioglycolate (TG) medium or LPS to induce acute peritonitis. In addition to the higher levels of pro‐inflammatory cytokines released from *Otud1* ^−/−^ mice, we also observed that a greater number of neutrophils accumulates in *Otud1* ^−/−^ mice (Figure 4d,e). Neutrophil recruitment to inflamed sites requires changes of surface signaling molecules, including adhesion molecules.^[^ ^20^ ^]^ We thus hypothesized that the inhibitory effects of OTUD1 on the secretory machinery also contributed to the distribution of adhesion molecules. To this end, we stimulated bone marrow derived neutrophils from WT and *Otud1* ^−/−^ mice by LPS and isolated cell membrane. Following quantitative proteomic analysis, we noticed that the majority of surface proteins were upregulated in OTUD1 deficient neutrophils and proteins associated with integrins, CD9 and CD47, were among these (Figure 4f). Subsequent flow cytometry assay confirmed that loss of OTUD1 facilitated CD9 and CD47 surface expression in neutrophils during periodontitis and peritonitis (Figure 4g,h; Figure S7A,B; Supporting Information). Consistent with these data in vivo, the cell surface expression levels of CD9 and CD47 were also enhanced in *Otud1* ^−/−^ neutrophils upon LPS or LPS‐PG treatment in vitro (Figure 4i,j; Figure S7C,D; Supporting Information). Notably, the total amounts of CD9 and CD47 were identical between WT and *Otud1* ^−/−^ neutrophils (Figure 4i,j; Figure S7C,D; Supporting Information). Collectively, our data indicate that OTUD1 inhibits neutrophil recruitment and effector function by limiting cellular secretory machinery.

### 2.6 OTUD1 Targets the COPII Complex and Suppresses the Cell Secretory Machinery

**Figure 5:**
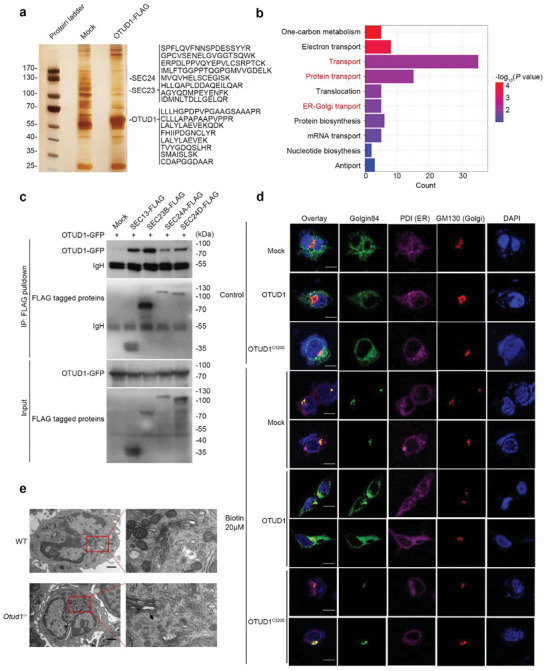
OTUD1 interacts with COPII components and inhibits protein transport. a) Mass spectrometry analysis of OTUD1‐associated proteins. HL‐60 cells stably expressing Mock or OTUD1‐FLAG were treated with ATRA (1 µM) and LPS (100 ng ml^−1^). FLAG‐tagged proteins were enriched by anti‐FLAG M2 beads, followed by mass spectrometry to identify the interactome of OTUD1. Matched peptides corresponding to OTUD1 and SEC23B are shown on the right panel. b) Enrichment analysis of the interactome of OTUD1 versus Mock in HL‐60 cells by GO databases (Fisher's Exact test, one‐sided, adjustments were not made for multiple comparisons) c) HEK293T cells were transfected with indicated plasmids and were subjected to immunoprecipitation with anti‐FLAG antibody, followed by western blot analysis. d) Imaging of cells expressing Ii‐streptavidin and Golgin 84‐EGFP‐SBP in HEK293T cells expressing Mock, OTUD1 or OTUD1^C320S^. PDI and GM130 were used to label the ER and Golgi apparatus, separately. Images were taken at 30 min after biotin (20 µM) treatment. The scale bars represent 10 µm. e) Representative pictures of neutrophils isolated from WT and *Otud1* ^−/−^ mice with LPS stimulation for 4 h obtained from transmission electron microscopy (scale bars represent 1 µm).

To investigate the molecular mechanism by which OTUD1 suppresses neutrophil secretion, we generated HL‐60 cell lines with stable expression of mock and FLAG‐tagged OTUD1, and utilized all‐trans retinoic acid (ATRA) to induce differentiation of leukemic cells into granulocytes. Upon LPS stimulation, we performed pull‐down assays with FLAG‐tagged proteins coupled with mass spectrometry to identify the interactome of OTUD1 (Figure S8A, Supporting Information). Interestingly, OTUD1 pulled down a group of mutually interacting transport proteins, including COPII components, SEC23 and SEC24 (**Figure** **5**a,b). Interaction between OTUD1 and COPII subunits was confirmed by co‐immunoprecipitation assay, and OTUD1 associated strongly with SEC23B (Figure 5c).

Since OTUD1 is a deubiquitinase, we therefore investigated the possibility that OTUD1 modulated SEC23B post‐translational modification. In vivo ubiquitination assay revealed that ectopic expression of OTUD1 rather than its inactive enzyme mutant C320S reduced SEC23B ubiquitination (Figure S8B, Supporting Information). Consistently, ubiquitination of endogenous SEC23B was increased in LPS‐elicited peritoneal neutrophils isolated from *Otud1* ^−/−^ mice (Figure S8C, Supporting Information).

The COPII complex functions in protein transport from the ER to Golgi apparatus. We next used the retention using selective hooks (RUSH) system to detect protein transport efficiency (Figure S8D, Supporting Information), and observed delayed protein transport in OTUD1 overexpressing cells, rather than OTUD1^C320S^ overexpressing cells (Figure 5d, Figure S8E,F, Supporting Information). Subsequent Transmission electron microscopy (TEM) observed that OTUD1 deficient neutrophils exhibited more pronounced transport vesicles trafficking, compared to WT neutrophils (Figure 5e). Collectively, these results imply that OTUD1 inhibits protein transport and ensuing surface molecule presentation via its deubiquitinating activity.

### 2.7 OTUD1 Removes the K63‐Linked Polyubiquitin Chain on SEC23B Lysine 81

**Figure 6:**
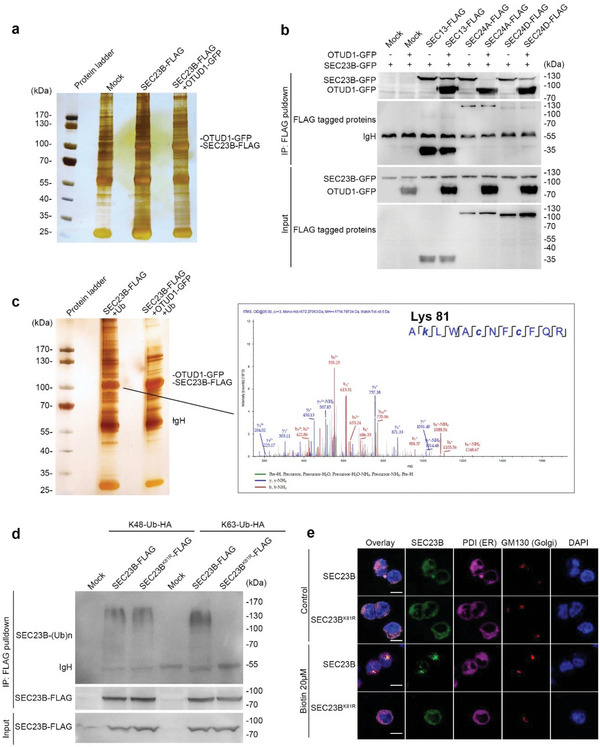
OTUD1 removes K63‐linked polyubiquitin chain on SEC23B Lysine 81. a) Mass spectrometry analysis of SEC23B‐associated proteins in the presence or absence of OTUD1. The bands indicating SEC23B and OTUD1 are shown. b) HEK293T cells were co‐transfected with vectors encoding FLAG tagged proteins and SEC23B‐GFP, with or without OTUD1‐GFP. After transfection, cell lysates were immunoprecipitated with FLAG antibody and protein A/G agarose beads and analyzed by immunoblot with anti‐GFP antibody. c) MS analysis of ubiquitination of SEC23B Lysine 81. SEC23B‐FLAG was co‐transfected with or without ectopically expressing OTUD1‐GFP, and then purified with anti‐FLAG M2 affinity gel. After sliver staining of the gel, mass spectrometry analysis was performed. d) In vivo ubiquitination assay of SEC23B. Lysates from HEK293T cells transfected with expression plasmids for variant SEC23B‐FLAG and indicated ubiquitin Lysine48‐only or Lysine 63‐only plasmids were subjected to immunoprecipitation with anti‐FLAG antibody followed by western blot analysis with anti‐HA antibody. e) Imaging of cells expressing Ii‐streptavidin and SEC23B‐EGFP‐SBP or SEC23B^K81R^‐EGFP‐SBP in HEK293T cells. The ER and Golgi apparatus were indicated with staining for PDI and GM130, separately. Images were taken at 30 min after biotin (20 µM) treatment. The scale bars represent 10 µm.

Further study showed that SEC23B underwent both K48‐ and K63‐linked polyubiquitination, which can be removed by OTUD1 (Figure S9A, Supporting Information). Given that K48‐linked ubiquitination is associated with proteasome‐mediated degradation of target substrates, we therefore treated cells with the protein synthesis inhibitor cycloheximide (CHX) to evaluate the effects of OTUD1 on SEC23B stability. As shown in Figure S9B (Supporting Information), the half‐life of SEC23B was similar in cells in presence or absence of OTUD1. Because K63‐linked ubiquitination is involved in the regulation of signal transduction, we therefore investigated whether OTUD1 affected the SEC23B interactome by mass spectrometry (**Figure** **6**a). The results showed that the presence of OTUD1 dampened the association of SEC23B with other COPII components, such as SEC13, SEC31A and SEC24A (Figure S9C, Supporting Information), which was confirmed by the co‐immunoprecipitation assay (Figure 6b). Using mass spectrometry, SEC23B ubiquitination was identified on Lysine 81 (K81), and this ubiquitination was blocked by OTUD1 (Figure 6c). By transfection plasmid encoding the Lysine 81 to Arginine (K81R) mutation of SEC23B, we determined that K81 on SEC23B was primarily conjugated with the K63‐linked polyubiquitin chain (Figure 6d). In order to ascertain the role of K63‐linked polyubiquitination on SEC23B‐mediated biological process, we applied anti‐FLAG pull‐down assay and found that the K81R mutant suppressed SEC23B interaction with SEC13 and SEC24D (Figure S9D,E, Supporting Information). Furthermore, we also used the RUSH system to monitor the protein transport process in cells with WT and K81R mutation of SEC23B. GFP‐tagged WT SEC23B was distributed in the ER, Golgi apparatus and cytoplasm after biotin was added. However, the K81R mutant SEC23B was predominantly sustained in the ER (Figure 6e; Figure S9F,G, Supporting Information). Together, these results thus demonstrate that the deubiquitinase OTUD1 limits the protein transport process by removing K63‐linked polyubiquitination of SEC23B.

### 2.8 Inhibition of Protein Secretory Signaling Alleviates Periodontal Immunopathology Induced by OTUD1 Deficiency

**Figure 7:**
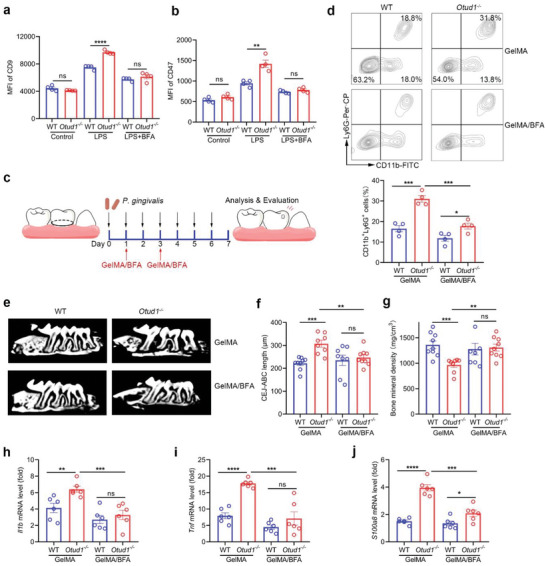
Blockade of the protein transport pathway attenuates periodontitis by OTUD1 deficiency. a) Flow cytometric analysis of surface CD9 expression level in bone marrow derived neutrophils in WT and *Otud1* ^─/─^ mice upon LPS (100 ng ml^−1^) and BFA (3 µg ml^−1^) treatment (n = 4, mean ± s.e.m., ns, not significant (*P* > 0.05), ****P = 0.00004, two‐tailed unpaired Student's t‐test). b) Flow cytometric analysis of surface CD47 expression level in bone marrow derived neutrophils in WT and *Otud1* ^─/─^ mice upon LPS (100 ng ml^−1^) and BFA (3 µg ml^−1^) treatment (n = 4, mean ± s.e.m., ns, not significant (*P* > 0.05), **P = 0.0029, two‐tailed unpaired Student's t‐test). c) Schematic illustration of BFA‐loading GelMA (GelMA/BFA) treatment. Experimental periodontitis was induced by ligature placement and *Porphyromonas gingivalis* (*P. gingivalis*) infection and the palatal gingivae of the second molars were injected with GelMA/BFA on day 1 and day 3. d) Flow cytometric analysis of neutrophils (CD11b^+^Ly6G^+^ cells) in WT and *Otud1* ^─/─^ mice gingiva after periodontitis induction and GelMA/BFA treatment (n = 4, mean ± s.e.m., *P = 0.0272, ***P = 0.0006 (GelMA, WT versus *Otud1* ^─/─^), ***P = 0.0008 (*Otud1* ^─/─^, GelMA versus GelMA/BFA), two‐tailed unpaired Student's t‐test). e) Representative micro‐CT sagittal images of maxillary alveolar bone surrounding the second molar after periodontitis induction and GelMA/BFA treatment. f) Micro‐CT analysis of CEJ‐ABC distance of second molar of WT or *Otud1* ^─/─^ mice after periodontitis induction and GelMA/BFA treatment (WT‐GelMA, n = 9; *Otud1* ^─/─^‐GelMA, n = 8; WT‐GelMA/BFA, n = 8; *Otud1* ^─/─^‐GelMA/BFA, n = 9; mean ± s.e.m., ns, not significant (*P* > 0.05), **P = 0.0063, ***P = 0.0002, two‐tailed unpaired Student's t‐test). g) Micro‐CT analysis of bone mineral density (BMD) around the second molar of WT or *Otud1* ^─/─^ mice after periodontitis induction and GelMA/BFA treatment (WT‐GelMA, n = 9; *Otud1* ^─/─^‐GelMA, n = 8; WT‐GelMA/BFA, n = 8; *Otud1* ^─/─^‐GelMA/BFA, n = 9; mean ± s.e.m., ns, not significant (*P* > 0.05), **P = 0.0012, ***P = 0.0007, two‐tailed unpaired Student's t‐test). h–j) RT–qPCR analysis of indicated mRNA levels in the gingiva tissues of WT and *Otud1* ^─/─^ mice after periodontitis induction and GelMA/BFA treatment (n = 6, mean ± s.e.m., ns, not significant (*P* > 0.05), *P = 0.0247, **P = 0.0067, ***P = 0.0009 (h), ***P = 0.0004 (i), ***P = 0.0002 (j), ****P < 0.0001, two‐tailed unpaired Student's t‐test).

Brefeldin A (BFA) has been reported to be a Golgi‐disruptor and can block presentation of membrane proteins. To study whether BFA treatment can weaken the stimulatory effects of OTUD1 deficiency on neutrophil secretion, we used LPS to stimulate WT or *Otud1* ^−/−^ neutrophils in the presence or absence of BFA. As expected, we found that BFA treatment reversed the upregulation of surface CD9 and CD47 expression by OTUD1 deficiency upon LPS stimulation (**Figure** **7**a,b; Figure S10A,B, Supporting Information). To further confirm this result in vivo, we exploited BFA‐loading gelatin methacryloyl (GelMA) (GelMA/BFA), which is moldable and injectable (Figure S10C,D, Supporting Information). Periodontitis was induced in WT and *Otud1* ^−/−^ mice and the palatal gingivae of the treated molars were injected with GelMA/BFA on day 1 and day 3 (Figure 7c). Administration of GelMA/BFA markedly suppressed neutrophil recruitment to the gingiva in *Otud1* ^−/−^ mice (Figure 7d). Furthermore, periodontal bone loss and inflammatory conditions were ameliorated effectively with GelMA/BFA injection (Figure 7e–j; Figure S10E–G, Supporting Information). Collectively, our data demonstrate that blocking protein transport signaling by BFA limits OTUD1‐deficient neutrophil secretion, eventually attenuating periodontal inflammation.

## 3 Discussion

Previous studies reported that OTUD1 mutations occurred in patients with auto‐inflammatory diseases, which was related to type‐I interferon signaling. However, the biological functions of OTUD1 would vary, depending on the specific tissues/organs and pathological conditions. Here, we report that deubiquitinase OTUD1 is selectively expressed in gingival neutrophils and upregulated during periodontitis. By triggering SEC23B deubiquitination, OTUD1 blocks the association with COPII components and protein trafficking, which in turn suppresses CD9/CD47‐mediated neutrophil recruitment to the inflamed foci and secretion of inflammatory cytokines. Reciprocally, loss of OTUD1 enhances neutrophil secretion and increases susceptibility to periodontitis (Figure S10H, Supporting Information). Our study therefore sheds light on the yet uncharacterized role of OTUD1 signaling in the modulation of neutrophil functions.

In addition to phagocytotic activity, neutrophils emerge to act as essential communicators that play crucial roles in shaping tissue microenvironment.^[^ ^5^ ^]^ For instance, neutrophils can initiate the adaptive immune responses by directly interacting with T cells.^[^ ^21^ ^]^ Neutrophils can also directly interact with dendritic cells (DCs), to limit the antigen presenting capacity of DCs, thereby affecting the efficacy of vaccination.^[^ ^22^ ^]^ It is critical to identify the regulators that control this secretory phenotype of neutrophils. Here we identified a subset of neutrophils with marked secretory phenotype, which displayed more pronounced cell‐cell communication with other immune cells. Most notably, loss of OTUD1 not only increases the percentage of secretory neutrophils, but also enhances signaling pathways related to the cellular secretory machinery. Moreover, activation of secretory signaling in neutrophils by OTUD1 depletion maintains the cell surface expression of integrins, which promotes neutrophil adhesion, chemotaxis and pro‐inflammatory cytokine release. Our data thus uncover the crucial role of OTUD1 signaling in the modulation of secretory neutrophil development.

As an essential COPII component, SEC23B functions in COPII assembly and vesicle budding. Loss of SEC23B has been demonstrated to be lethal in mice because of pancreatic insufficiency and hypoglycaemia.^[^ ^23^ ^]^ Biallelic mutations in the *SEC23B* gene (20p11.23) cause type II congenital dyserythropoietic anemia (CDAII) due to aberrant assembly or deconstruction of the midbody during cytokinesis and erythroid differentiation disorder.^[^ ^24^, ^25^ ^]^ Post‐translational modification plays a key role in the regulation of SEC23B. In response to starvation, UNC51‐like kinase 1 (ULK1) phosphorylates SEC23B on Serine 186, which inhibits F‐box protein FBXW5‐mediated SEC23B degradation, thereby promoting autophagic flux.^[^ ^26^ ^]^ In this study, we found that ubiquitination of SEC23B was increased during LPS stimulation, which can be restrained by OTUD1 overexpression. Through mass spectrometry analysis, we found that OTUD1 targets SEC23B and removes the K63‐linked polyubiquitin chain of SEC23B, which blocks its association with COPII subunits. Moreover, we showed that Lysine 81 of SEC23B can be modified by the K63‐linked polyubiquitin chain. The K81R mutant inhibits SEC23B ubiquitination and attenuates SEC23B‐mediated CD9/CD47 surface expression. OTUD1 therefore exerts its suppressive function upon neutrophil migration through blockade of SEC23B ubiquitination.

Trafficking of transmembrane and secretory proteins from the ER to Golgi apparatus is mediated by COPII vesicle, which is composed of secretion associated Ras related GTPase 1 (SAR1), SEC23‐SEC24 heterodimeric complex, and SEC13‐SEC31 heterodimeric complex.^[^ ^27^, ^28^ ^]^ Emerging evidence have now shown that dysregulation of COPII components is associated with host immune disorders. SEC23B‐deficient T cells have been observed to exhibit accumulation of secreted proteins, proliferative defects and reduced effector functions.^[^ ^29^ ^]^ SEC24A participates in the ER‐to‐Golgi transport of programmed death ligand 1 (PD‐L1) and stabilizes Golgi residency of PD‐L1, thereby maintaining PD‐L1 expression in tumor cells and resistance to immunotherapy.^[^ ^30^ ^]^ The wet and highly dynamic environment of the oral cavity often makes local treatment of periodontitis challenging. To overcome this, we employed photo‐cross‐linking of GelMA loaded with BFA for tight adhesion and longer duration. We observed that blockade of ER‐to‐Golgi transport by BFA effectively inhibited expression of surface molecules and neutrophil migration to the gingiva, thereby ameliorating periodontal inflammation and alveolar bone resorption, thus suggesting BFA treatment as an adjuvant therapy for periodontitis.

Periodontitis is a prevalent chronic dental disease in which a polymicrobial infection of anaerobic gram‐negative bacteria in the sub‐gingival tissue, triggering a deregulated host immune response that leads to the breakdown of the periodontal ligament and alveolar bone.^[^ ^31^ ^]^ In particular, persistent inflammatory stimulation in periodontal tissues leads to alveolar bone loss around the teeth. To further investigate the pathogenesis of periodontitis, the experimental mouse periodontitis model induced by *P. gingivalis* and ligature placement was developed that provides an accelerated pathogenesis of periodontitis, including predictable bone loss, periodontal tissue damage and the early innate immune elements that characterize chronic periodontitis.^[^ ^32^ ^]^ However, it is possible that this rapid disease induction may not completely mimic the chronic properties of human chronic periodontitis. The biological function of OTUD1 in tissue remodeling and adaptive immune response during chronic periodontitis thereby needs further investigation.

In conclusion, our data demonstrate that OTUD1 acts as a self‐regulator of periodontal inflammation. Upon neutrophil activation, OTUD1 expression is induced and limits SEC23B K63‐ubiquitination, which in turn restricts protein transport and blocks the secretory neutrophil polarization, eventually ameliorating tissue inflammatory damage. Our findings thus shed new light on an unexplored mechanism of neutrophil‐mediated periodontitis, implying that OTUD1 or protein transport signaling may be a potential target for periodontitis treatment.

## 4 Experimental Section

### Mice

*Otud1* ^─/─^ mice have been described previously.^[^ ^17^ ^]^ All animals were maintained in a special pathogen‐free environment. All sex‐and age‐matched animal experimental procedures have been approved by the ethics committee of Peking University Health Science Center (approved number PUIRB‐LA2022608).

### Patients and Specimens

Inclusion criteria for healthy subject were: ≥ 18 years of age, no symptoms of periodontal inflammation and without history of infection of HBV, HCV, HIV, and syphilis, active malignancy, autoimmune disorders and use of systemic antibiotics. Inclusion criteria for periodontitis group were: clinical attachment loss (CAL) ≥ 5 mm and periodontal probing depth (PPD) ≥ 6 mm and significant inflammation signs including gingival erythema and bleeding upon probing. ≈4 mm × 2 mm buccal gingival biopsies were obtained under local anesthesia and periodontal ligament tissues were obtained from the extracted teeth of patients. All procedures were carried out under the approval of the Ethics Committee of Peking University School and Hospital of Stomatology (approved number PKUSSIRB‐202281139), and the informed consent was obtained from all subjects (in accordance with the Helsinki Declaration).

### Induction of Experimental Periodontitis

Experimental periodontitis was induced in mice by 5‐0 silk ligation and 10^9^ CFU *P. gingivalis* (ATCC 33 277) infection to the maxillary second molar. After 1 week, mice maxilla specimen was fixed with 4% (w/v) paraformaldehyde and analyzed using a computed micro‐tomography X‐ray (micro‐CT) 3D imaging system (Y. Cheetah, YXLON International GmbH, Germany) at 60 kV, and 220 µA. The images were reconstructed and analyzed by the Inveon Research Workplace software. The distance from CEJ to ABC and bone mineral density (BMD) was measured to evaluate the alveolar bone resorption.

### Induction of Acute Peritonitis

To create the thioglycollate (TG)‐induced acute peritonitis model, 6‐week‐old male WT and *Otud1* ^─/─^ mice were injected intraperitoneally with 1 ml of 3% (w/v) TG medium (Sigma, T9032) and elicited cells were harvested after 2.5 h by peritoneal lavage with 10 ml of pre‐cold PBS. For LPS‐induced peritonitis model, 6‐week‐old male WT and *Otud1* ^─/─^ mice were injected intraperitoneally with 20 mg kg^−1^ LPS (Sigma, L2880), and elicited cells were harvested after 4 h.

### Bone Marrow Transplantation

Recipient mice were lethally irradiated (1000 cGy mouse^−1^), and intravenously injected with 5 × 10^6^ bone marrow cells from donor mice femurs and tibias. Periodontitis induction on the transplanted mice were performed after 2 months to ensure that bone marrow reconstitution had occurred.^[^ ^33^ ^]^

### Co‐Immunoprecipitation and Immunoblot Analysis

HEK293T cells were transfected with the indicated plasmids and lysed by lysis buffer containing 10% (v/v) glycerol, 0.5% (v/v) NP‐40, 150 mM NaCl, and 0.1 mM EDTA with protease inhibitor cocktail (Roche). Cell lysates were incubated with anti‐FLAG antibody (Sigma, F3165) and protein A/G (Santa Cruz Biotechnology, sc‐2003). The beads were then washed three times by PBSN (PBS containing 0.1% NP‐40) and subjected to SDS‐Page. Antibodies against OTUD1 (homemade),^[^ ^14^ ^]^ Ub (FK2) (LifeSensors, LSS‐AB120), SEC23B (homemade),^[^ ^34^ ^]^ FLAG (Sigma, F3165), GFP (RayAntibody, RM1008), HA (Sigma, H3663) and GAPDH (RayAntibody, RM2002) were used in this study.

### Preparation of Immune Cells

For isolation of gingiva infiltrating immune cells, gingiva tissues were incubated with digestion solution containing 0.5 mg mL^−1^ collagenase D (Roche, 11 088 866 001) and 0.1 mg mL^−1^ DNase I (Sigma, DN25) at 37 °C. After filtration, CD45^+^ immune cells were isolated by a BD FACS Aria II flow cytometer. Bone marrow derived neutrophils were isolated by Percoll gradients. WT and *Otud1* ^─/─^ mice bone marrow was collected from femurs and tibias by perfusion cold PBS. Bone marrow cells were then pelleted by centrifugation and resuspended in PBS. Percoll solutions were diluted with PBS to obtain gradients of 72%, 60%, and 52%. Bone marrow cell suspensions were loaded in the uppermost layer. The neutrophils were then collected at between the 60% and 72% layers.

### In Vivo Ubiquitination Assay

For in vivo deubiquitination assays, HEK293T cells were transfected with FLAG‐SEC23B, GFP‐OTUD1, and HA‐Ub. At 24 h later, the cells were lysed with co‐immunoprecipitation lysis buffer and were subjected to affinity purification with anti‐FLAG M2 beads. After washing with PBSN for three times, the binding components were analyzed by Western blot assay. To detect ubiquitination of endogenous SEC23B in neutrophils, the bone marrow derived neutrophils were isolated and stimulated with LPS (100 ng ml^−1^) for 4 h. Neutrophils were then lysed and anti‐SEC23B antibody was used to enrich endogenous SEC23B. The ubiquitination of SEC23B was then analyzed by anti‐Ub antibody.

### Protein Half‐Life Assay

For the SEC23B half‐life assay, HEK293T cells were transfected with FLAG‐OTUD1 and treated with the protein synthesis inhibitor cycloheximide (Biorbyt, 100 µg mL^−1^) for the indicated time durations before collection. Endogenous SEC23B was then detected by anti‐ SEC23B antibody.

### Flag Pulldown Assay

HL‐60 cells stably expressing Mock or OTUD1‐FLAG were treated with ATRA (1 µM) to induce differentiation of leukemic cells into granulocytes for 5 days and then stimulated by LPS (100 ng ml^−1^) for 4 h. FLAG‐tagged proteins were enriched by anti‐FLAG M2 beads (Sigma, F2426) and the binding components were eluted with 3×FLAG peptide (Sigma, F4799). The samples were subjected to NuPAGE 4%−12% gel (Invitrogen) and sliver staining (Pierce, 24 612). The excised gel segments were subjected to mass spectrometry to identify the interactome of OTUD1. To identify ubiquitination sites of SEC23B, FLAG‐SEC23B was transfected in HEK293T cells with or without GFP‐OTUD1. FLAG‐SEC23B was enriched as mentioned above. After sliver staining of the gel, excised gel segments were subjected to in‐gel trypsin digestion and dried.

### Quantitative Proteomic Analysis

Bone marrow derived neutrophils from WT and *Otud1* ^─/─^ mice were treated by LPS (100 ng ml^−1^) or LPS‐PG (1 µg ml^−1^) for 4 h, and the cell membrane protein or cell culture supernatant was isolated and subjected to mass spectrometry analysis.

### Mass Spectrometry Analysis

Peptides were dissolved in 0.1% (v/v) formic acid and the samples were eluted for 50 min with linear gradients of 5–32% acetonitrile in formic acid, at a flow rate of 300 nl min^−1^. Mass spectrometry data were acquired with LTQ Orbitrap Elite mass spectrometer (Thermo Fisher Scientific) equipped with a nanoelectrospray ion source (Proxeon Biosystems). The raw files were searched with the SEQUEST engine against a database from the UniProt protein sequence database.

### Single Cell RNA‐Sequencing

CD45^+^ immune cells were isolated from gingiva by a BD FACS Aria II flow cytometer. 20000 cells were barcoded and pooled using the 10× Genomics device. Samples were prepared following the manufacturer's protocol and sequenced on an Illumina NextSeq sequencer. To produce a raw unique molecular identifier (UMI) count matrix, the raw data were aligned and quantified using the Cell Ranger Single‐Cell Software Suite (version 5.0.0, 10× Genomics) against the mm10 reference genome. The matrix was converted into a Seurat object by the R package Seurat software (version 3.2.3). For quality control, cells with over 15% mitochondrial‐derived UMI counts were removed and the remaining 17 995 single cells were applied in downstream analyses, separately. The UMI count matrix was normalized with the SCTransform function. Then, integration on two datasets was performed. In this process, potential anchors were created with FindIntegrationAnchors function of Seurat using the top 3000 variable genes, and IntegrateData function was used to integrate data and create a new matrix, in which the potential batch effect was regressed out. To reduce the dimensionality of the scRNA‐Seq dataset, principal component analysis (PCA) was performed. The top 40 PCs were used to perform the downstream analysis with Elbowplot function. Subsequently, uniform manifold approximation and projection (UMAP) were performed. The Seurat FindClusters function was used to divide all cells into several clusters with resolution set at 0.5, and the Seurat FindAllMarkers function was performed to identify preferentially expressed genes within clusters. The Seurat FindMarkers function was used to identify differentially expressed genes between the two samples in each group. The R package dplyr software (version 1.0.3) was used to count the proportion of each cell type. The CellChat was used to infer cell‐cell communications between neutrophils with other immune cells.

### H&E (Hematoxylin‐eosin) Staining and IHC (immunohistochemistry)

For H&E staining, the tissues were fixed with formaldehyde and then embedded with paraffin. Sections of 4 µm thickness were used for H&E staining with a standard protocol.^[^ ^35^ ^]^ Images were acquired using an Olympus IX51 microscope. For detecting the expression of OTUD1 and S100A8/S100A9 in gingiva, the sections were dehydrated with graded concentrations of ethanol and endogenous peroxidase activity was blocked with 3% (v/v) hydrogen peroxide in methanol for 10 min. Then, antigen retrieval was carried out using 1 mM of EDTA buffer (PH 9.0) in a microwave oven. The sections were then incubated with rabbit anti‐human polyclonal OTUD1 (abcam, ab122481) or anti‐S100A8/S100A9 (abcam, ab288715). Sections were developed with the Envision Detection System (Dako) and counterstained with haematoxylin.

### Retention using Selective Hooks (RUSH)

RUSH assay was performed as previously reported.^[^ ^36^ ^]^ In brief, HEK293T cells expressing Mock, OTUD1 or OTUD1^C320S^ were transfected with Ii‐streptavidin and Golgin 84‐EGFP‐SBP, followed by seeding on cover glass. After 24 h, biotin (20 µM or 40 µM) was added for 30 min and the cells were fixed with 4% (w/v) paraformaldehyde, permeabilized with 0.5% (v/v) Triton X‐100 and blocked by 1% (w/v) BSA, followed by staining with specific antibodies anti‐PDI (Abcam, ab2792), anti‐GM130 (Abclonal, A5433) and DAPI. Images were acquired with a Nikon TCS A1 microscope.

### Transmission Electron Microscopy (TEM)

WT and *Otud1* ^─/─^ neutrophils were stimulated with LPS for 4 hours and fixed with 4% glutaraldehyde for 12 h, post‐fixed with 2% OsO4 for 2 h, and then dehydrated. The fixed samples were subsequently cut into thin sections (60 nm). The sections were placed on formvar coated slot grids, post‐stained in uranyl acetate and Reynold's lead citrate and imaged on TEM (Hitachi, H‐600‐II).

### Flow Cytometry

To analyze cell surface maker expression, the cells were incubated with specific antibodies for 30 min at room temperature. The following antibodies were used: anti‐CD45 (eBioscience, 30‐F11), anti‐CD11b (Biolegend, M1/70), anti‐Ly6G (eBioscience, RB6‐8C5), anti‐CD9 (Biolegend, MZ3) and anti‐CD47 (Biolegend, miap301).

### Preparation of BFA‐Loading GelMA

Brefeldin A (BFA) was dissolved in 10% (w/v) gelatin methacryloyl (GelMA) (EFL‐GM‐30) at a concentration of 100 µg ml^−1^. Periodontitis was induced in WT and *Otud1* ^−/−^ mice and the palatal gingivae of the treated molars were injected with 10 µl GelMA/BFA on day 1 and day 3, followed by exposure to a blue light source with a wavelength of 405 nm.

### Quantitative Real‐Time PCR

Total RNA was extracted with Trizol reagent (Invitrogen) and then reverse transcribed into cDNA according to the manufacturer's protocol (Vazyme, R223‐01). The cDNA quantification was performed using an ABI 7500 Detection System (all primers, Table S1, Supporting Information).

### Enzyme‐Linked Immunosorbent Assay (ELISA)

Enzyme‐linked immunosorbent assays for IL6, IL1β, TNFα and IFNγ in mouse serum were performed according to the manufacturer's instructions (IL6, R&D, M6000B; IL‐1β: R&D, MLB00C; TNFα: R&D, MTA00B; IFNγ: R&D, MIF00).

### Secretory Cytokine Detection

Bone marrow derived neutrophils from WT or *Otud1* ^─/─^ mice were isolated and stimulated with LPS (100 ng ml^−1^) for 4 h. Cell culture supernatant was collected and secretory cytokines were measured by Proteome Profiler Mouse Cytokine Array Kit (R&D Systems, ARY006) according to the manufacturer's instructions.

### Routine Blood Test

Experimental periodontitis was induced in WT and *Otud1* ^−/−^ mice by ligature placement and *P. gingivalis* infection. The inner canthus blood was collected and subjected to routine blood tests using a HEMAVET 950FS Veterinary Multi‐species Hematology System (Drew scientific), following the manufacturer's instruction.

### Statistical Analysis

Statistical analysis was performed using the Prism GraphPad software v7.0. Differences between the two groups were calculated using a two‐tailed Student's t test. *P < *0.05 was considered significant.

## Conflict of interest

The authors declare no conflicts of interest.

## Author Contributions

J.S. and Y.Z. contributed equally to this work. X.D., D.L., and Xuehui. Z. conceived the study. J.S. and Y.Z. performed most of the experiments and analyzed the data. X.S., Y.G., and Y.L. performed the animal experiments. Y.B., Y.H., M.G., X.C., M.X., and Y.W. provided human samples. Xin. Z and W.L. performed bioinformatics analysis. F.Y. provided animal model. J.S., D.L., X.D., B.C.H. and Xuehui. Z. wrote the paper.

## Supporting information

Supporting Information

## Data Availability

The data that support the findings of this study are available from the corresponding author upon reasonable request.
