# Supplementary material for: The Deubiquitinase OTUD1 Suppresses Secretory Neutrophil Polarization And Ameliorates Immunopathology of Periodontitis
Source: Adv Sci (Weinh). 2023 Aug 28;10(30):2303207. doi: 10.1002/advs.202303207 (PMC10602526; doi:10.1002/advs.202303207)
Supplement: Supplementary file 1 — Supporting Information [file ADVS-10-2303207-s001.pdf]

ADVANCED SCIENCE
Open Access

Supporting Information

for *Adv. Sci.*, DOI 10.1002/advs.202303207

The Deubiquitinase OTUD1 Suppresses Secretory Neutrophil Polarization And Ameliorates Immunopathology of Periodontitis

Jia Song, Yuning Zhang, Yunyang Bai, Xiaowen Sun, Yanhui Lu, Yusi Guo, Ying He, Min Gao, Xiaopei Chi, Boon Chin Heng, Xin Zhang, Wenjing Li, Mingming Xu, Yan Wei, Fuping You, Xuehui Zhang*, Dan Lu* and Xuliang Deng**

Supporting Information

The deubiquitinase OTUD1 suppresses secretory neutrophil polarization and ameliorates immunopathology of periodontitis

Jia Song, Yuning Zhang, Yunyang Bai, Xiaowen Sun, Yanhui Lu, Yusi Guo, Ying He, Min Gao, Xiaopei Chi, Boon Chin Heng, Xin Zhang, Wenjing Li, Mingming Xu, Yan Wei, Fuping You, Xuehui Zhang, Dan Lu, Xuliang Deng

Figure S1

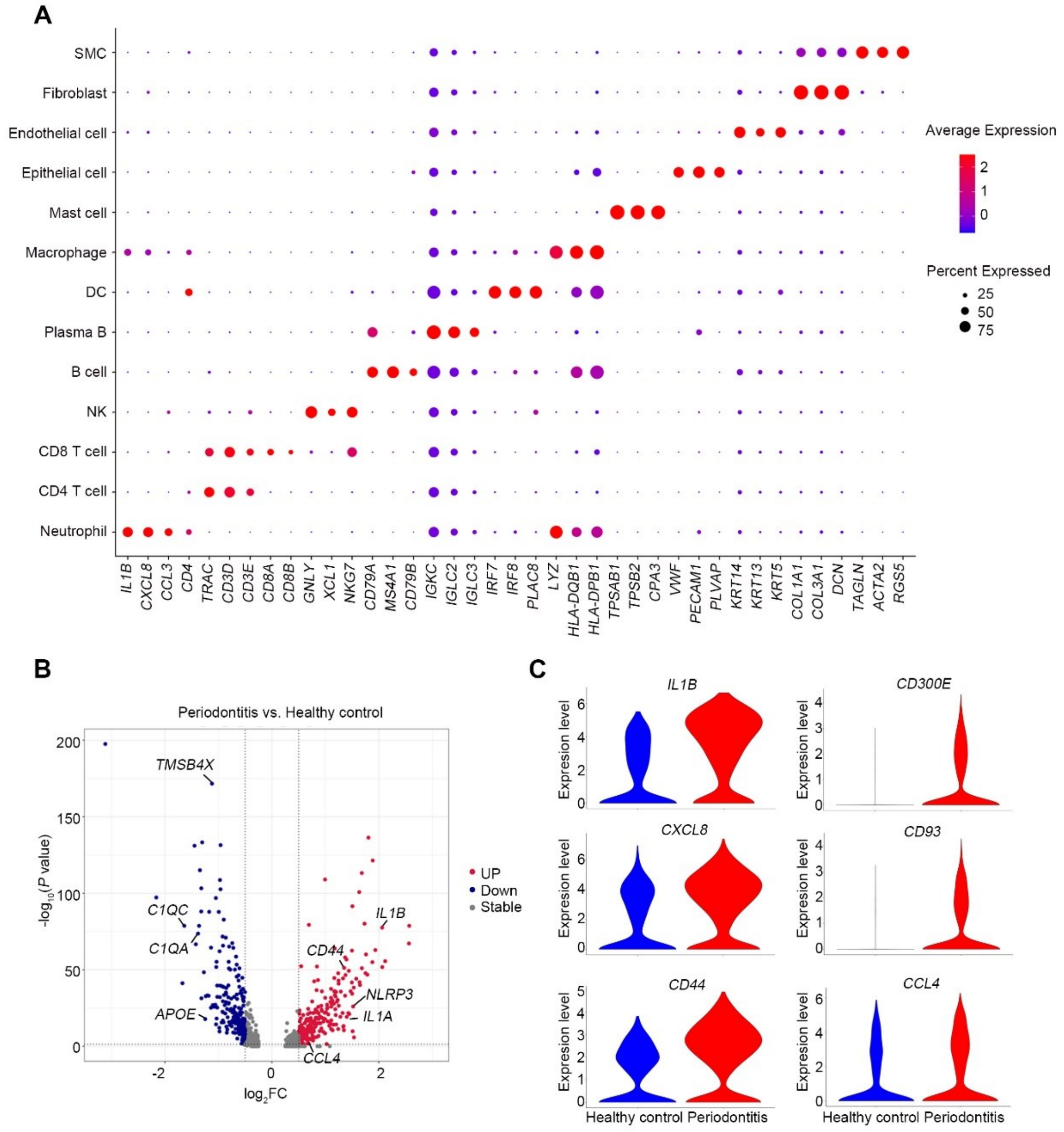

Figure S1. Single-cell RNA-seq analysis of the characteristic of neutrophils in patients with periodontitis and healthy subjects.

A) Heatmap plot showing the expression levels of known cell-type-enriched marker genes across 13 cell clusters. The color intensity indicates the normalized expression of marker genes in each cell population. The size of the dot represents the cell proportion that express the marker gene.

B) Volcano plots analysis of the differentially expressed genes (DEGs) in neutrophils

between patients with periodontitis and healthy control. Red, genes upregulated in neutrophils of patients with periodontitis vs. healthy subjects. Blue, genes downregulated in neutrophils of patients with periodontitis vs. healthy subjects.

C) Violin plots analysis of the differentially expressed genes in neutrophils between patients with periodontitis and healthy control.

Figure S2

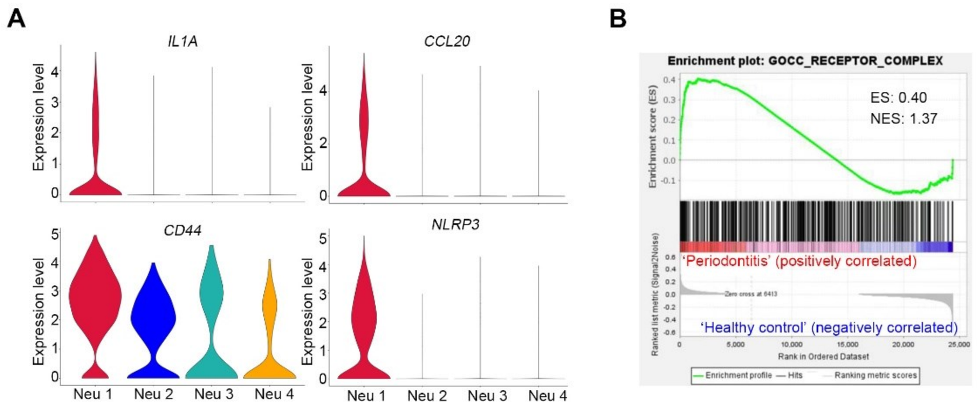

Figure S2. Single-cell RNA-seq analysis of the characteristic of neutrophil subpopulations in patients with periodontitis and healthy subjects.

A) Violin plots analysis of the differentially expressed genes in 4 neutrophils clusters.

B) GSEA of genes expressed in Neu 1 of patients with periodontitis and healthy subjects.

ES, enrichment score; NES, normalized enrichment score.

Figure S3

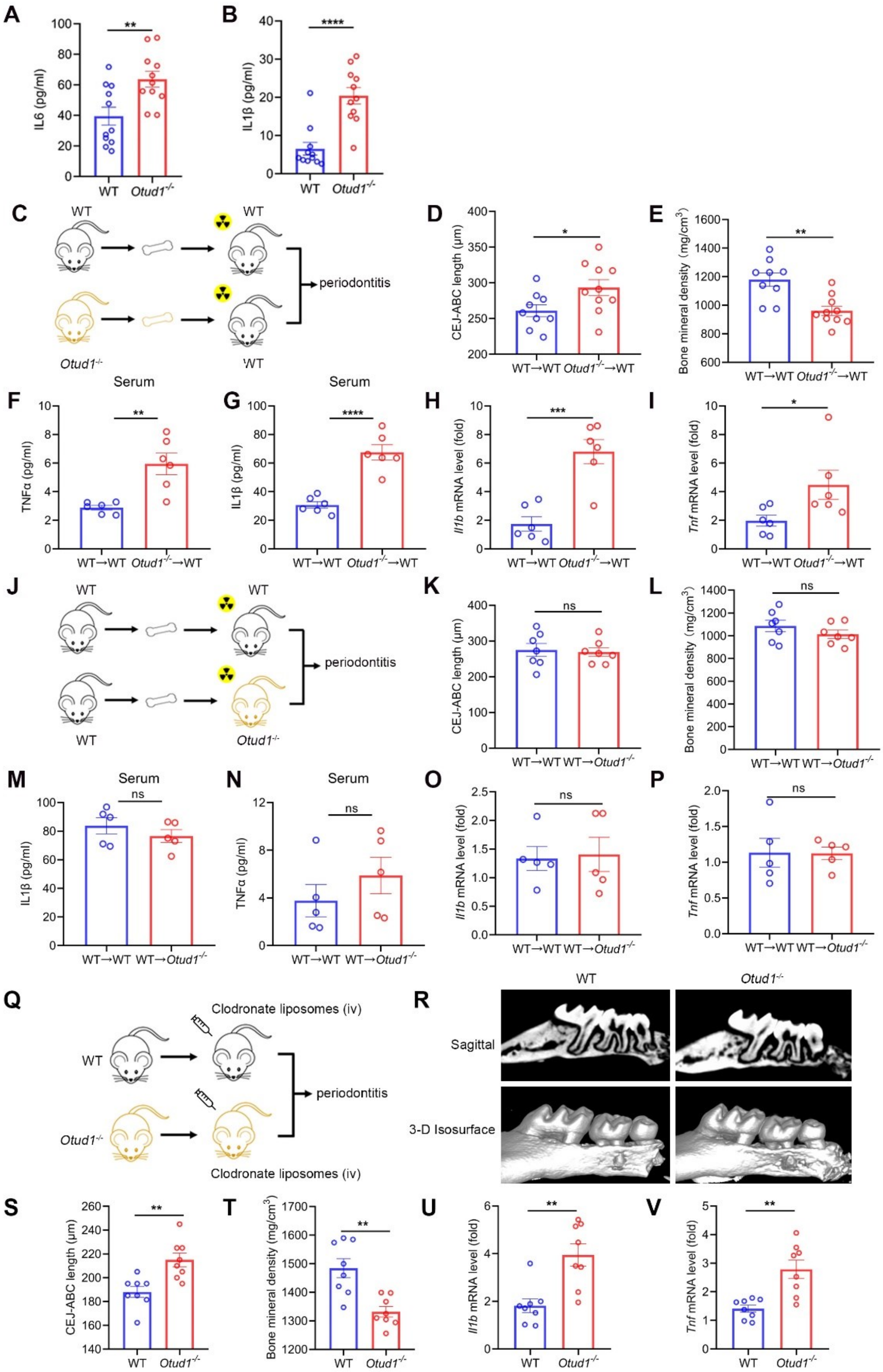

Figure S3. OTUD1 plays a key role in neutrophil during periodontitis.

A, B) Protein expression levels of indicated pro-inflammatory cytokines within mice serum in the context of experimental periodontitis (n = 11, mean ± s.e.m., ***P* = 0.0058,

****$P = 0.00005$, two-tailed unpaired Student's t-test).

C) A schematic illustration of the bone marrow transplantation assay. WT mice were lethally irradiated and received bone marrow cells from WT or *Otud1*$^{-/-}$ mice. Experimental periodontitis induction was performed after bone marrow reconstitution.

D) Micro-CT analysis of CEJ-ABC distance of the second molar of WT mice that received WT or *Otud1*$^{-/-}$ mice bone marrow (WT→WT, n = 9; *Otud1*$^{-/-}$→WT, n = 10; mean ± s.e.m., *$P = 0.0359$, two-tailed unpaired Student's t-test).

E) Bone mineral density (BMD) around the second molar of WT mice that received WT or *Otud1*$^{-/-}$ mice bone marrow was analyzed by micro-CT imaging (WT→WT, n = 9; *Otud1*$^{-/-}$→WT, n = 10; mean ± s.e.m., **$P = 0.0012$, two-tailed unpaired Student's t-test).

F, G) Protein expression levels of indicated pro-inflammatory cytokines within mice serum in the context of experimental periodontitis (n = 6, mean ± s.e.m., **$P = 0.0025$, ****$P = 0.00008$, two-tailed unpaired Student's t-test).

H, I) RT–qPCR analysis of indicated pro-inflammatory cytokine mRNA levels in gingiva tissues of WT mice that received bone marrow from WT and *Otud1*$^{-/-}$ mice after periodontitis induction (n = 6, mean ± s.e.m., *$P = 0.0430$, ***$P = 0.0004$, two-tailed unpaired Student's t-test).

J) A graphic model of bone marrow transplantation assay. WT and *Otud1*$^{-/-}$ mice were lethally irradiated and received bone marrow cells from WT mice. Experimental periodontitis induction was performed after bone marrow reconstitution.

K) Analysis of CEJ-ABC distance around second molar from WT or *Otud1*$^{-/-}$ mice

received WT mice bone marrow obtained by micro-CT imaging (n = 7; mean ± s.e.m., ns, not significant ($P > 0.05$), two-tailed unpaired Student's t-test).

L) Analysis of bone mineral density (BMD) around second molar from WT or *Otud1*$^{-/-}$ mice received WT mice bone marrow obtained by micro-CT imaging (n = 7; mean ± s.e.m., ns, not significant ($P > 0.05$), two-tailed unpaired Student's t-test).

M, N) Protein levels of indicated pro-inflammatory cytokines in mice serum in the context of experimental periodontitis (n = 5, mean ± s.e.m., ns, not significant ($P > 0.05$), two-tailed unpaired Student's t-test).

O, P) RT–qPCR analysis of indicated pro-inflammatory cytokine mRNA levels in gingiva tissues from WT and *Otud1*$^{-/-}$ mice received bone marrow from WT mice after periodontitis induction (n = 5, mean ± s.e.m., ns, not significant ($P > 0.05$), two-tailed unpaired Student's t-test).

Q) A schematic illustration of the macrophage depletion assay. WT or *Otud1*$^{-/-}$ mice were intravenously injected with clodronate liposomes to delete macrophages. Experimental periodontitis induction was then performed.

R) Representative micro-CT sagittal and 3-D Isosurface images of maxillary alveolar bone surrounding the second molar after periodontitis induction in the context of macrophage depletion.

S, T) Micro-CT analysis of CEJ-ABC distance and bone mineral density (BMD) around the second molar from WT or *Otud1*$^{-/-}$ mice with macrophage depletion (n = 8; mean ± s.e.m., $^{**}P < 0.01$, two-tailed unpaired Student's t-test).

U, V) RT–qPCR analysis of *Il1β* and *Tnf* mRNA levels in gingival tissues from WT and

Otud1$^{-/-}$ mice with macrophage depletion after periodontitis induction (n = 8, mean ± s.e.m., $^{**}P < 0.01$, two-tailed unpaired Student's t-test).

Figure S4

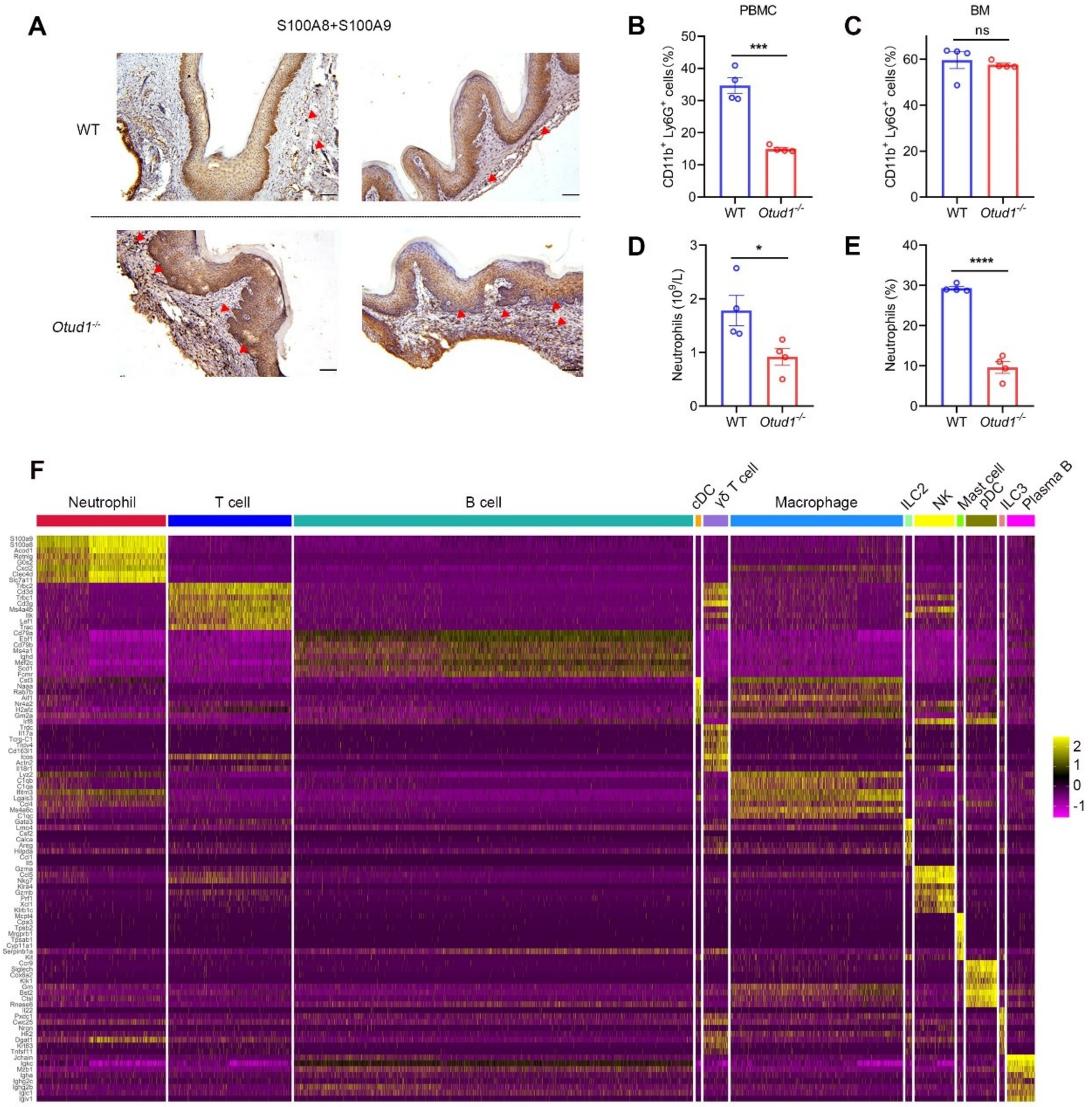

Figure S4. Loss of OTUD1 promotes neutrophil recruitment to inflamed foci.

A) Representative immunohistochemistry staining pictures of S100A8/S100A9 protein levels in gingiva tissues from WT and *Otud1*$^{-/-}$ mice after periodontitis induction. The scale bars represent 100 μm.

B, C) Flow cytometric analysis of neutrophil ($CD11b^{+}Ly6G^{+}$ cell) in WT and *Otud1*$^{-/-}$ peripheral blood mononuclear cell (PBMC) and bone marrow (BM) after periodontitis induction (n = 4, mean ± s.e.m., ns, not significant ($P > 0.05$), ****P* = 0.0002, two-tailed unpaired Student's t-test).

D, E) Quantities of neutrophils in blood from WT and *Otud1*$^{-/-}$ mice were analyzed by routine blood test using a HEMAVET 950FS Veterinary Multi-species Hematology System (n = 4, mean ± s.e.m., *P = 0.0366, ****P = 0.00002, two-tailed unpaired Student's t-test).

F) Heatmap plot showing the expression levels of known cell-type-enriched marker genes across 12 cell clusters.

Figure S5

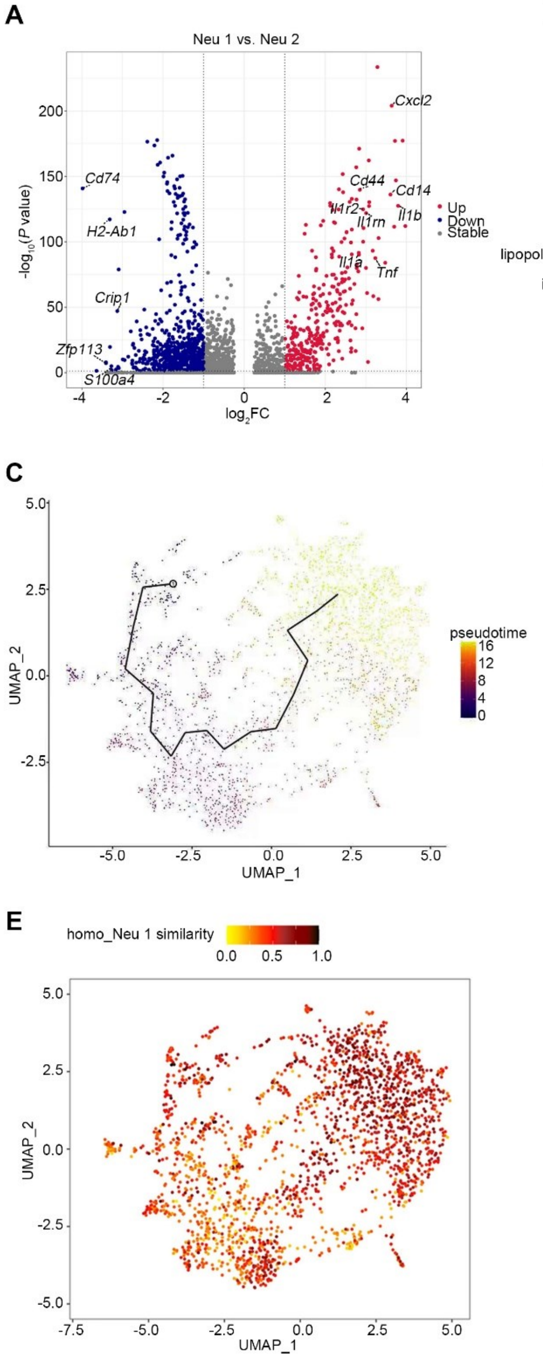

Figure S5. Single-cell RNA-seq analysis of the characteristic of neutrophil subpopulations.

A) Volcano plots analysis of the differentially expressed genes (DEGs) between Neu 1 and Neu 2. Red, genes upregulated in Neu 1 vs. Neu 2. Blue, genes downregulated in Neu 1 vs. Neu 2.

B) Genes upregulated in Neu 1 were analyzed with Gene Ontology (GO_BP) terms.

C) RNA velocity streamline plot of UMAP from the scRNA-seq experiment reveals neutrophil-lineage commitment.

D, E) Similarity of neutrophil cell clusters from mice and Neu1 derived from patients with periodontitis and healthy subjects analyzed by AUcell.

Figure S6

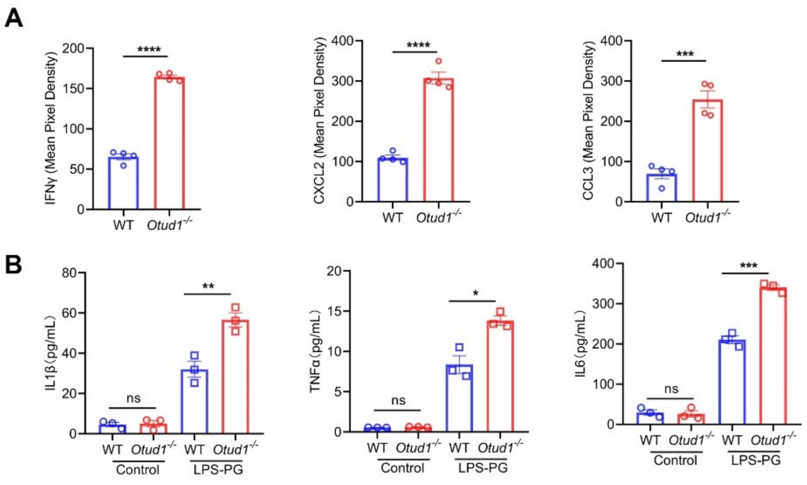

Figure S6. OTUD1 deficiency enhances the secretion of effector cytokines of neutrophils.

A) Quantitative analysis of average relative expression levels of indicated secretory cytokines (n = 4, mean ± s.e.m., ***P = 0.0003, ****P < 0.0001, two-tailed unpaired Student's t-test).

B) Cell culture supernatant of neutrophils from WT and *Otud1*$^{-/-}$ mice upon LPS-PG (1 µg/ml) stimulation was collected and secretory proinflammatory cytokines were measured by ELISA assay (n = 3, mean ± s.e.m., ns, not significant (P > 0.05), *P = 0.0116, **P = 0.0092, ***P = 0.0004, two-tailed unpaired Student's t-test).

Figure S7

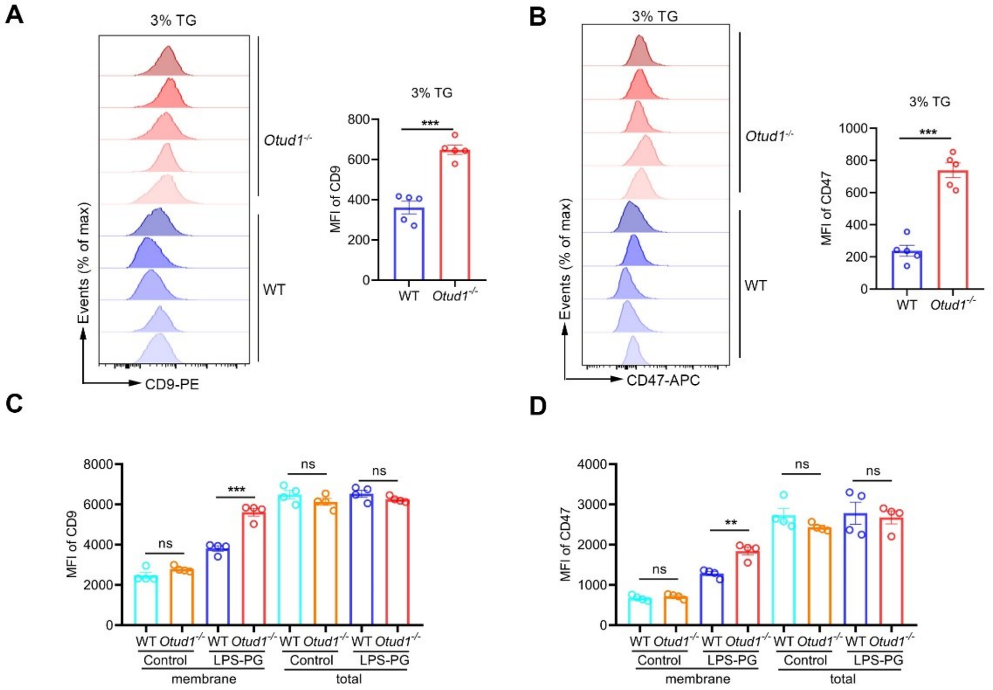

Figure S7. Loss of OTUD1 promotes expression of CD9 and CD47 on the cell surface of neutrophils.

A) Flow cytometric analysis of CD9 expression level in intraperitoneal neutrophils in WT and *Otud1*$^{-/-}$ mice after peritonitis induction by TG medium (n = 5, mean ± s.e.m., ***P = 0.00007, two-tailed unpaired Student's t-test).

B) Flow cytometric analysis of CD47 expression level in intraperitoneal neutrophils in WT and *Otud1*$^{-/-}$ mice after peritonitis induction by TG medium (n = 5, mean ± s.e.m., ***P = 0.00002, two-tailed unpaired Student's t-test).

C, D) Flow cytometric analysis of membrane or total CD9/CD47 expression level in bone marrow derived neutrophils in WT and *Otud1*$^{-/-}$ mice upon LPS-PG (1 μg/ml) stimulation (n = 4, mean ± s.e.m., ns, not significant ($P > 0.05$), **P = 0.0022, **P = 0.0003, two-tailed unpaired Student's t-test).

Figure S8

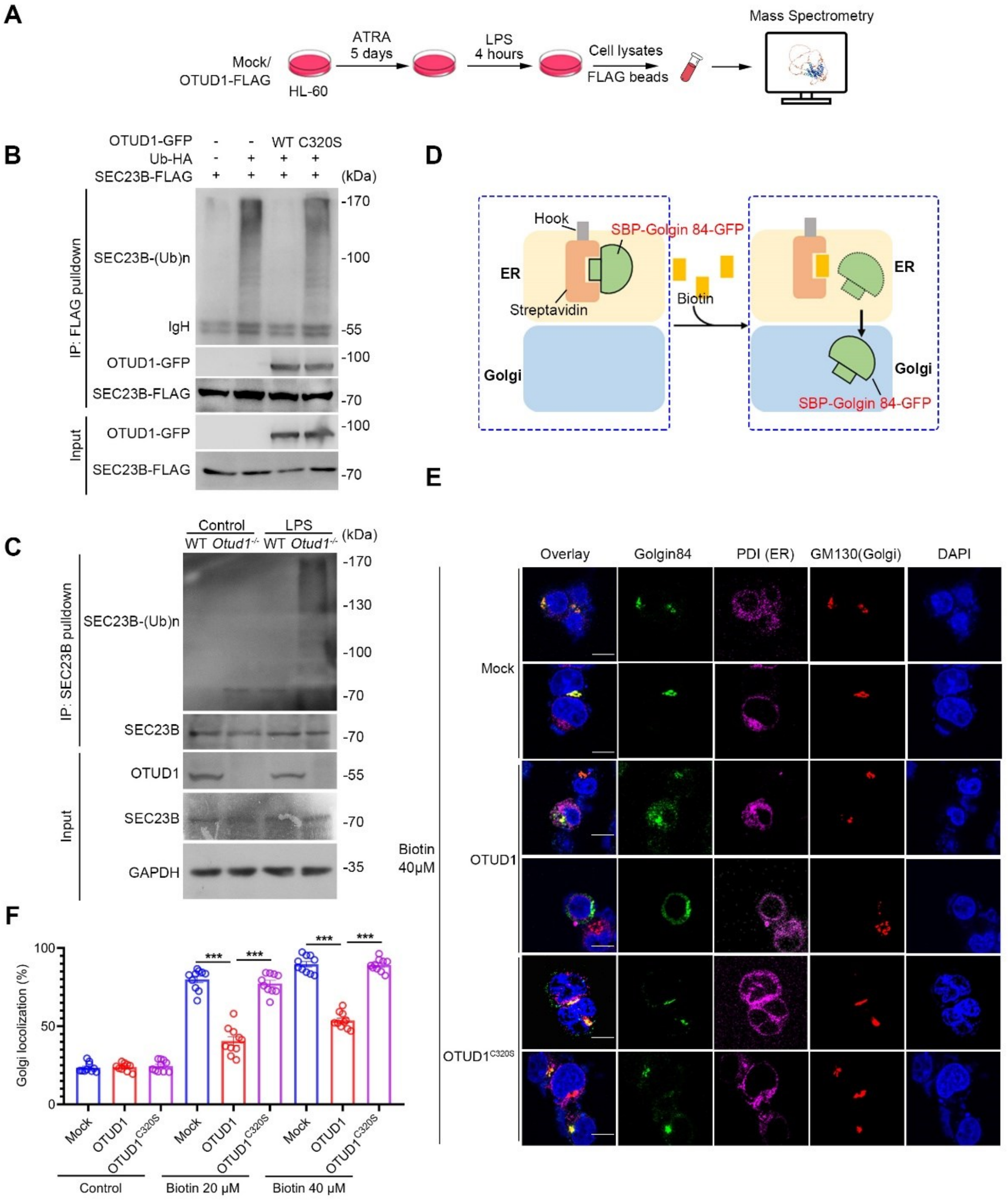

Figure S8. OTUD1 targets SEC23B and inhibits ER-to-Golgi trafficking.

A) Workflow of MS to identify OTUD1-associated proteins. Mock or FLAG tagged OTUD1 stably expressed HL-60 cell lines were generated and treated by ATRA (1 μM) for 5 days to induce differentiation of leukemic cells into granulocytes. After LPS (100 ng/ml) stimulation for 4 hours, FLAG-tagged proteins were enriched by anti-FLAG M2

beads and followed by MS.

B) *In vivo* ubiquitination assay of SEC23B. HEK293T cells were transfected with indicated plasmids and subjected to immunoprecipitation with anti-FLAG antibody followed by western blot analysis.

C) *In vivo* ubiquitination assay of SEC23B. Bone marrow derived neutrophils were isolated with WT and $Otud1^{-/-}$ mice and treated by LPS (100 ng/ml) for 4 hours. Neutrophils were lysed, endogenous SEC23B was immunoprecipitated by anti-SEC23B antibody and subsequently analyzed by anti-ubiquitin antibody for the assessment of ubiquitination.

D) A graphic model of retention using selective hooks (RUSH) system to detect protein transport efficiency. In brief, plasmid expressing Ii-streptavidin and Golgin 84-EGFP-SBP was transfected in HEK293T cells. Golgin 84-EGFP-SBP is retained in ER via its interaction with the hook. This interaction is mediated by streptavidin and streptavidin binding protein (SBP). After biotin added, Golgin 84-EGFP-SBP was released and trafficked to Golgi apparatus.

E) Imaging of cells expressing Ii-streptavidin and Golgin 84-EGFP-SBP in HEK293T cells expressing Mock, OTUD1 or $OTUD1^{C320S}$. PDI and GM130 were used to label ER and Golgi apparatus, separately. Pictures were taken at 30 min after biotin (40 μM) treatment. The scale bars represent 10 μm.

F) Statistic analysis of Golgin 84 Golgi localization after biotin supplement by ImageJ (n = 10 cells, mean ± s.e.m., $***P < 0.0001$, two-tailed unpaired Student's t-test).

Figure S9

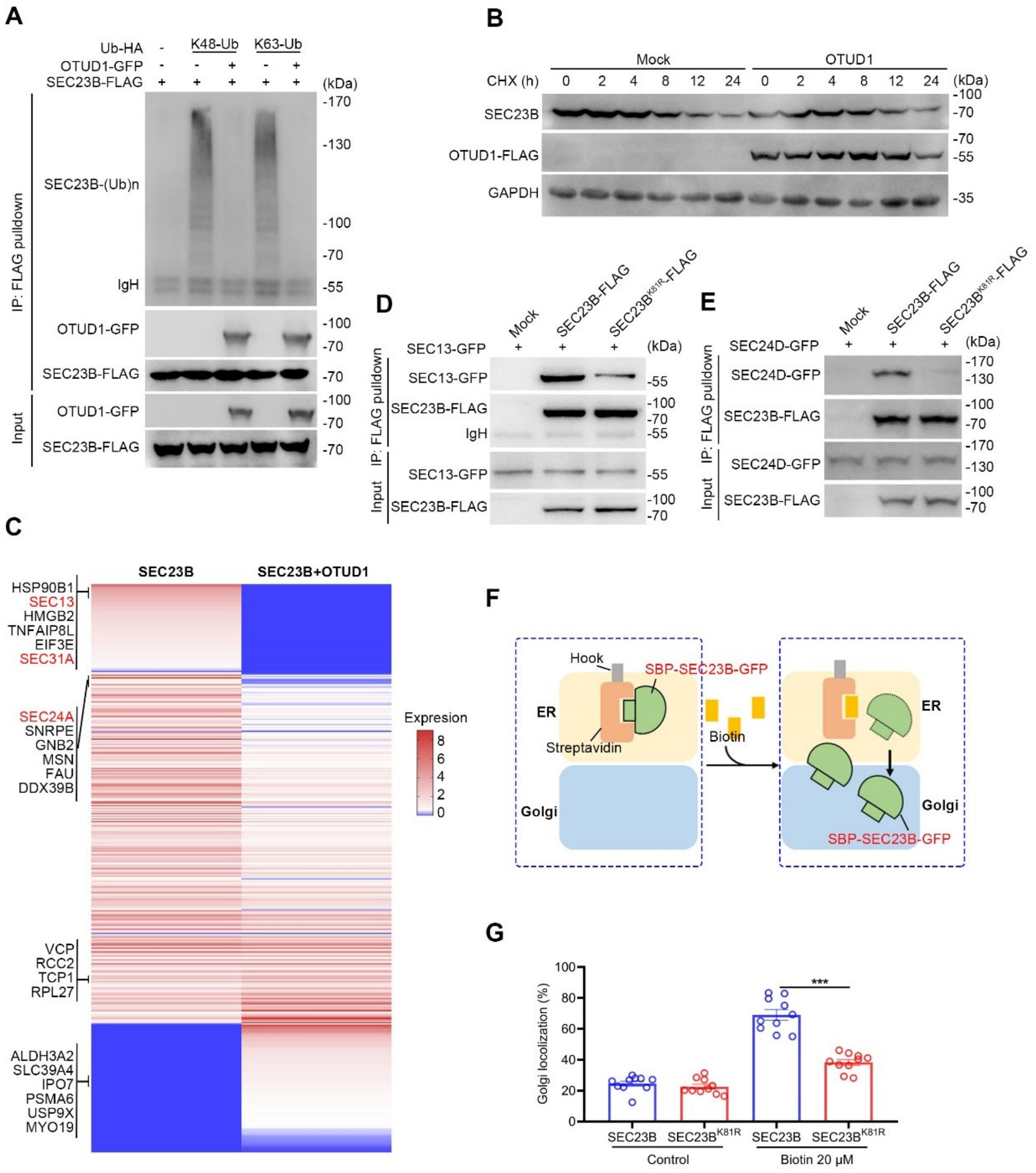

Figure S9. OTUD1 suppresses protein transport by deubiquitinating SEC23B Lysine 81.

A) *In vivo* ubiquitination assay of SEC23B. Lysates from HEK293T cells transfected with expression plasmids for SEC23B-FLAG and indicated ubiquitin Lysine48-only or Lysine 63-only plasmids were subjected to immunoprecipitation with anti-FLAG antibody followed by western blot analysis with anti-HA antibody.

B) Half-life analysis of SEC23B in the presence or absence of OTUD1. Cells were treated with 100 μg/ml cycloheximide (CHX) and collected at the indicated times for western blot analysis.

C) Heatmap of the SEC23B associated proteins in the presence or absence of OTUD1 was shown.

D, E) HEK293T cells were transfected with indicated plasmids and were subjected to immunoprecipitation with anti-FLAG antibody followed analysis by western blot.

F) A graphic model of retention using selective hooks (RUSH) system to detect protein SEC23B transport efficiency. In brief, plasmid expressing Ii-streptavidin and SEC23B-EGFP-SBP or SEC23B^{K81R}-EGFP-SBP was transfected in HEK293T cells. SEC23B-EGFP-SBP is retained in ER via its interaction with the hook. This interaction is mediated by streptavidin and streptavidin binding protein (SBP). After biotin added, SEC23B-EGFP-SBP was released and subcellular localization of SEC23B or SEC23B^{K81R} was monitored.

G) Statistic analysis of SEC23B or SEC23B^{K81R} Golgi localization after biotin supplement by ImageJ (n = 10 cells, mean ± s.e.m., ****P* = 0.0000003, two-tailed unpaired Student's t-test).

Figure S10

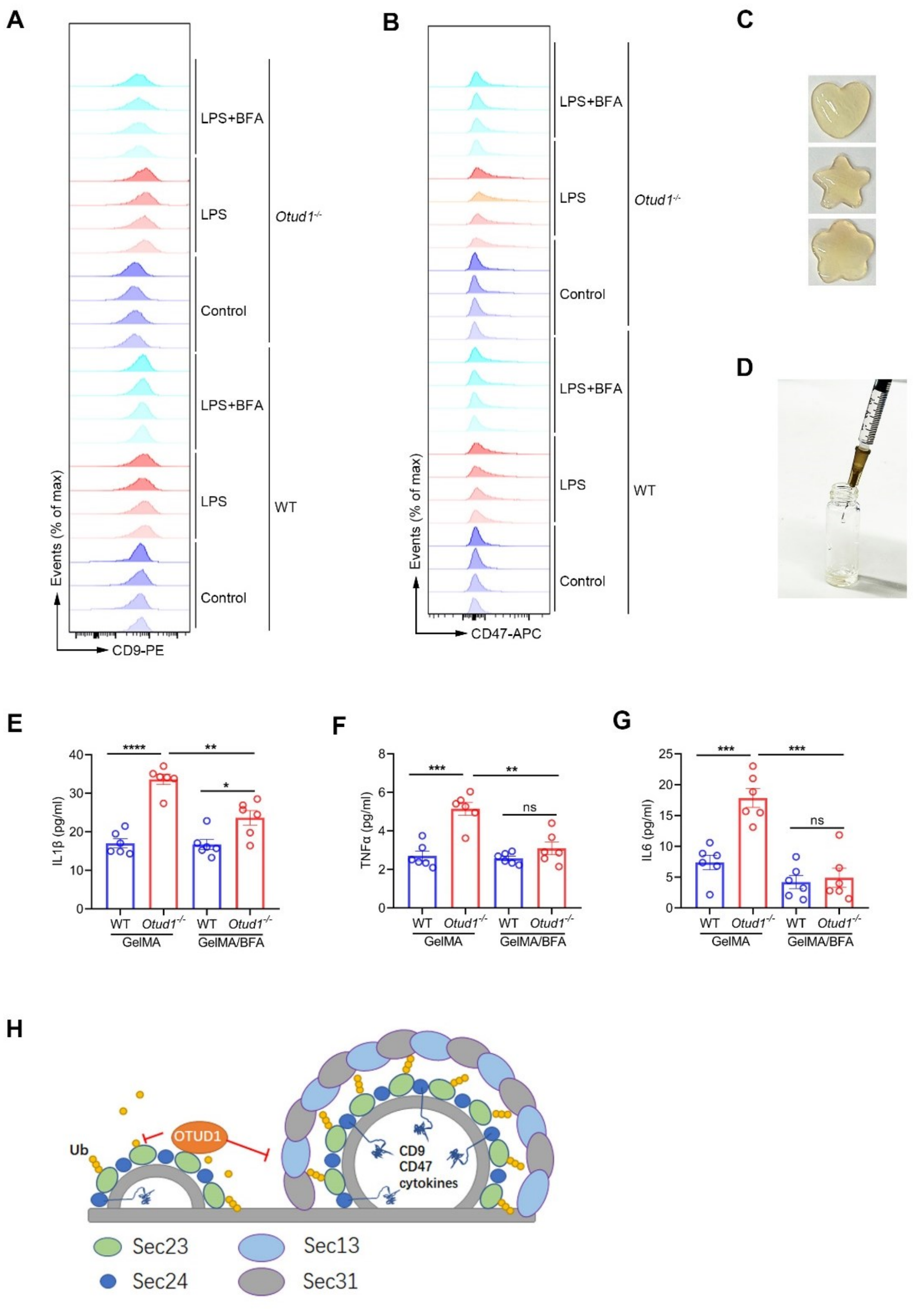

Figure S10. Blockade of protein transport by BFA limits secretory molecules expression in neutrophils.

A, B) Flow cytometric analysis of CD9 and CD47 surface expression in WT and *Otud1*$^{-/-}$ neutrophils with LPS (100 ng/ml) stimulation and BFA (3 µg/ml) treatment.

C) GelMA was quickly molded into various shapes via photo-crosslinking at 405 nm

light.

D) Photograph of gel of injectability.

E, F, G) Protein levels of indicated pro-inflammatory cytokines in mice serum in the context of experimental periodontitis (n = 6, mean ± s.e.m., ns, not significant (P > 0.05), *P = 0.0133, **P = 0.0015 (IL1β), **P = 0.0015 (TNFα), ***P = 0.0002 (TNFα), ***P = 0.0003 (IL6, WT vs. *Otud1*$^{-/-}$), ***P = 0.0001 (IL6, GelMA-*Otud1*$^{-/-}$ vs. GelMA/BFA-*Otud1*$^{-/-}$), ****P = 0.000003 (IL1β), two-tailed unpaired Student's t-test).

H) A graphic model illustrating the role of OTUD1 in modulation of neutrophil secretory phenotype. OTUD1 blocks SEC23B ubiquitination and inhibits its association between COPII components, thereby limiting ER-to-Golgi trafficking and sustaining periodontal immune homeostasis.

Table S1. The primers used for quantitative real-time PCR were as follows:

Primer name	Sequence (5'->3') -Forward	Sequence (5'->3') -Reverse
OTUD1	GATTCCCTGAGGCCTAGTATTT	CAGTTGTCGTACTCTGGGTTAG
Il6	TCTGCAAGAGACTTCCATCC	GAATTGCCATTGCACAACTC
Il1β	CAACCAACAAGTGATATTCTCC	TGCCGTCTTTCATTACACAG
Tnfα	CCCTCACACTCAGATCATCTTCT	GCTACGACGTGGGCTACAG
S100a8	AAATCACCATGCCCTCTACAAG	CCCACTTTTATCACCATCGCAA
Mpo	AGTTGTGCTGAGCTGTATGGA	CGGCTGCTTGAAGTAAAACAGG
Actb	GAGACCTTCAACACCCCAGC	ATGTCACGCACGATTTCCC
GAPDH	ACCCACTCCTCCACCTTTGA	CTGTTGCTGTAGCCAAATTCGT
